# Asymmetric gene expression and cell-type-specific regulatory networks in the root of bread wheat revealed by single-cell multiomics analysis

**DOI:** 10.1186/s13059-023-02908-x

**Published:** 2023-04-04

**Authors:** Lihua Zhang, Chao He, Yuting Lai, Yating Wang, Lu Kang, Ankui Liu, Caixia Lan, Handong Su, Yuwen Gao, Zeqing Li, Fang Yang, Qiang Li, Hailiang Mao, Dijun Chen, Wei Chen, Kerstin Kaufmann, Wenhao Yan

**Affiliations:** 1 National Key Laboratory of Crop Genetic Improvement, Hubei Hongshan Laboratory, Huazhong Agricultural University, Wuhan, 430070 China; 2 Wuhan Igenebook Biotechnology Co., Ltd, Wuhan, 430014 China; 3 State Key Laboratory of Pharmaceutical Biotechnology, School of Life Sciences, Nanjing University, Nanjing, 210023 China; 4 Department for Plant Cell and Molecular Biology, Institute for Biology, Humboldt-Universität Zu Berlin, 10115 Berlin, Germany

## Abstract

**Background:**

Homoeologs are defined as homologous genes resulting from allopolyploidy. Bread wheat, *Triticum aestivum*, is an allohexaploid species with many homoeologs. Homoeolog expression bias, referring to the relative contribution of homoeologs to the transcriptome, is critical for determining the traits that influence wheat growth and development. Asymmetric transcription of homoeologs has been so far investigated in a tissue or organ-specific manner, which could be misleading due to a mixture of cell types.

**Results:**

Here, we perform single nuclei RNA sequencing and ATAC sequencing of wheat root to study the asymmetric gene transcription, reconstruct cell differentiation trajectories and cell-type-specific gene regulatory networks. We identify 22 cell types. We then reconstruct cell differentiation trajectories that suggest different origins between epidermis/cortex and endodermis, distinguishing bread wheat from *Arabidopsis*. We show that the ratio of asymmetrically transcribed triads varies greatly when analyzing at the single-cell level. Hub transcription factors determining cell type identity are also identified. In particular, we demonstrate that TaSPL14 participates in vasculature development by regulating the expression of *BAM1*. Combining single-cell transcription and chromatin accessibility data, we construct the pseudo-time regulatory network driving root hair differentiation. We find MYB3R4, REF6, HDG1, and GATAs as key regulators in this process.

**Conclusions:**

Our findings reveal the transcriptional landscape of root organization and asymmetric gene transcription at single-cell resolution in polyploid wheat.

**Supplementary Information:**

The online version contains supplementary material available at 10.1186/s13059-023-02908-x.

## Background

Polyploidy species possess multiple sets of genomes that originated from whole-genome duplication (WGD) or interspecific hybridization [1–3]. Polyploidization is regarded as a driver for plant speciation, evolution, and biodiversity [4–7]. Increasing evidence shows a correlation between polyploidization and the potential for adaptation to environmental stress [5, 7–9]. Polyploidization causes the changes of genome and epigenome organization, as well as the restructurings of transcriptome, metabolome, and proteome [8]. Asymmetric expression of homoeologs is prevalent in polyploidy, which renders the dominant loci controlling specific agronomic traits in specific subgenome [3, 10–12]. However, our current understanding toward asymmetric expression of homoeologs is based on data from a particular tissue or organ, which is a mixture of multiple cell types. The lack of a reference single-cell transcriptional atlas obscures the details of asymmetric expression caused by cellular heterogeneity.

Recently, application of single-cell RNA sequencing (scRNA-seq) in plants improved the understanding of cell heterogeneity in various plant species, such as *Arabidopsis*, rice, maize, cotton, peanut, and poplar [13–29]. In *Arabidopsis*, rice and maize, the root cell types, have been well profiled [14, 17, 18, 20–22]. Transcriptome heterogeneity can be compared in rice and *Arabidopsis* root by scRNA-seq. The results showed that two special cell clusters of sclerenchyma and exodermis existed in rice [22], but absent in *Arabidopsis* [21]. The sclerenchyma and exodermis can prevent radial oxygen loss in rice roots in paddy field [22]. The root differentiation of monocots is distinct from that in dicots, such that the lateral root cap and epidermis of monocots do not share the common progenitor cells as in the dicot model species *Arabidopsis* [22, 30, 31]. scRNA-seq technology enables the reconstruction of cellular differentiation trajectories and novel insights into organ development. The scRNA-seq on rice radicles revealed differentiation trajectories of exodermis, sclerenchyma, and cortex and the results showed that these ground tissues originated from the same meristem cell population [22]. ScRNA-seq analysis also led to generation of new hypotheses on gene function, for instance, Ortiz-Ramirez and colleagues demonstrated that the mobility of transcription factor SHORT-ROOT (SHR) is necessary for cortical layer division in maize [18].

Bread wheat (*Triticum aestivum* L., AABBDD, 2n = 6x = 42) is allohexaploid and evolved from the interspecific hybridizations of three distinct diploid species, *Triticum urartu* (AA), *Aegilops speltoides* (BB), and *Aegilops tauschii* (DD) contributing the AA, BB, and DD genome, respectively [3, 32–35]. Asymmetric expression of homoeologs may overcome the inter-genomic incompatibilities and conflicting transcriptional events [10]. Organ development requires the coordination of gene expression among three subgenomes. The heterologous polyploid genome and adaptation to its upland habitat may have contributed to a special root organization and transcriptome heterogeneity in wheat, compared to *Arabidopsis* and rice [31]. To comprehensively understand the asymmetric expression of homoeologs based on cell transcriptome heterogeneity and to decode cell-type-specific activities of regulatory elements in wheat root, we performed single-nuclei RNA sequencing (snRNA-seq) and single-nuclei assay for transposase-accessible chromatin with high-throughput sequencing (snATAC-seq). We also explored asymmetric gene expression pattern at single-cell resolution. Moreover, we constructed cell-type-specific networks controlling cell identity and identified novel TFs driving cell fate change from meristematic cells to root hair by combining cell-type-specific gene expression and chromatin accessibility. An online web server (http://crispr.hzau.edu.cn/wheataltas/html/destinations.html) was also provided for user to easily use and visualize the snRNA-seq and snATAC-seq datasets generated in this study.

## Results

### Generation of a single-cell transcriptome and regulome atlas of the wheat root

**Fig. 1:**
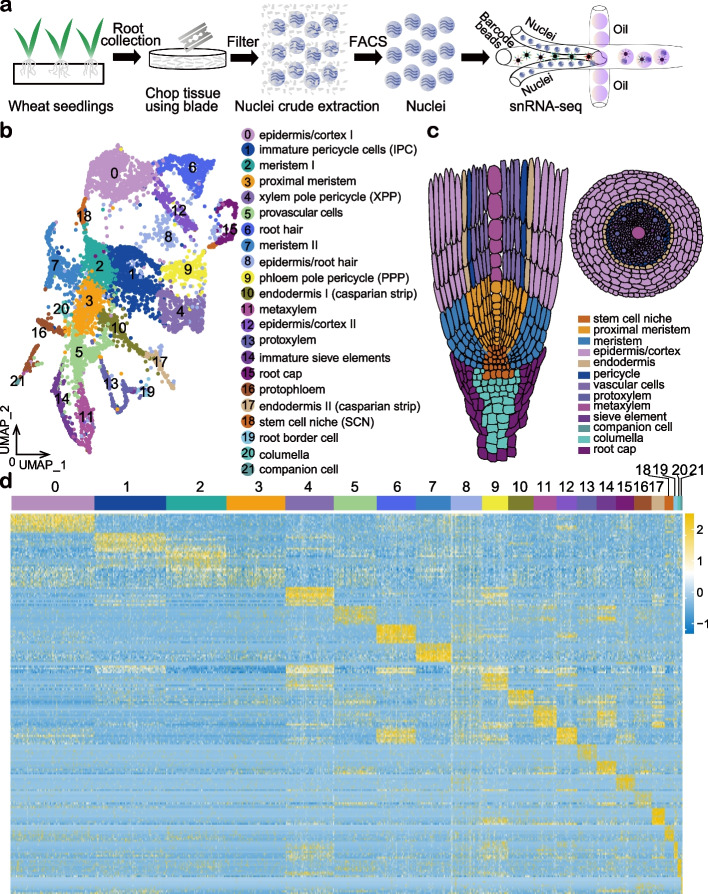
Cell types identified in wheat root tips based on snRNA-seq. **a** Nuclei based single-cell transcription analysis in wheat. **b** UMAP visualization of 22 cell clusters annotated for wheat root tips. Each dot represents a single cell. Colors of dots are corresponding to cell clusters. IPC, immature pericycle cells; XPP, xylem pole pericycle; PPP, ploem pole pericycle; SCN, stem cell niche. **c** Schematic diagram of wheat root in anatomy. **d** The expression patterns of representative top 10 cluster-specific marker genes in 22 clusters

It has been shown that protoplast isolation process generates side effects and the affected genes are highly variable with batches, which may affect cell clustering and cell type annotation [22, 36]. To eliminate the protoplasting effect, snRNA-seq has been applied in plant single-cell omics [37, 38]. Expression profiles generated from the plant nuclei are regarded as snapshots of the dynamic transcriptional activity of the genes and are comparable to the profiles from protoplasts [36, 37, 39]. We established a simple and effective single-nuclei RNA sequencing protocol with wheat root (Fig. 1a, “Methods” section). The 0.5-cm root tips of elite wheat variety Aikang58 (AK58) were harvested and chopped for nuclei isolation. High-quality nuclei were obtained by fluorescence-activated cell sorting (FACS) and about 15,000 nuclei were loaded into 10 × Chromium Chip B for snRNA-seq (Additional file 1: Fig. S1a). The result showed that snRNA-seq with nuclei sorting process greatly reduced pollution from mitochondrial (median < 0.005%) and chloroplast (median < 0.025%) (Additional file 1: Fig. S2a). The data produced by snRNA-seq and bulk RNA-seq were highly correlated (*R* = 0.78), which indicates the overall reliability of the snRNA-seq data (Additional file 1: Fig. S2b). A gene expression matrix containing 6875 cells was generated using the snRNA-seq data. In total, 78,763 genes were identified as expressed genes and on average, 4094 genes were detected per single nuclei (Additional file 2: Table S1), which was comparable with published single-cell datasets from *Arabidopsis* (Additional file 1: Fig. S2c and Additional file 2: Table S1) [21, 39].

To map the activities of *cis*-regulatory elements at single-cell resolution, we performed the snATAC-seq in wheat root. In total, 10, 000 intact nuclei were submitted to Tn5 tagmentation and then were loaded into 10 × Chromium Chip E for snATAC-seq (Additional file 1: Fig. S1b). An average of 38,628 reads per nuclei was obtained. For most of the cells, the score of transcription start site (TSS) enrichment per cell (average accessibility ratio of TSS position / TSS flanking positions, defined by the ArchR software) was greater than five (Additional file 1: Fig. S2d and S2e) and the median value of TSS enrichment score was 8.868. The unique nuclear fragments in most cells were greater than 1 × 10^3^, and the median fragments were 11,060 (Additional file 1: Fig. S2e). Quality control parameters indicated a high quality of the snATAC-seq data. Taken together, our results indicated a high quality of snRNA-seq and snATAC-seq data for wheat root.

### Identification of cell types in the wheat root

Since the functions of most genes in wheat are largely unknown, information from *Arabidopsis* was used to interpret the wheat data in the initial analysis. Homologous genes with high sequence similarity and comparable expression pattern were proposed to have similar function (Additional file 2: Table S2). *t*-distributed stochastic neighborhood embedding (*t*-SNE) tool and uniform manifold approximation and projection (UMAP) algorithm were independently used for cell clustering (Fig. 1b and Additional file 1: Fig. S3) [40, 41]. UMAP was found to depict the hierarchical structure of cell clusters more clearly than *t*-SNE [21]. Therefore, a UMAP scatter plot (Fig. 1b) and a schematic diagram of root anatomy (Fig. 1c) were present to show the annotated cell clusters and their locations within the root. To determine the spatial relationship among cell clusters, we also constructed the three-dimensional UMAP scatterplot for cell clusters (Additional file 3). The corresponding cluster-specific marker genes (Additional file 2: Table S2) were used for cell cluster annotation. In total, 22 clusters with biological significance were identified (Fig. 1b, Additional file 2: Table S2 and Additional file 4), which demonstrated a high heterogeneity of wheat root tips. The top 10 cluster-specific marker genes with specific expression pattern were shown in Fig. 1d. We then investigated the expression of long noncoding RNAs in the 22 clusters. Then, 50–125-long noncoding RNAs were identified in the particular clusters (Additional file 2: Table S3). Interestingly, we found cluster-specific marker long noncoding RNAs for clusters 15, 17, 18, 20, and 21 (Additional file 1: Fig. S4). The cell cluster annotation showed that the root stele was consisted of protophloem, protoxylem, metaxylem, sieve element, companion cell, and pericycle; the ground tissue was composed of root hair, epidermis/cortex, and endodermis; the root cap zone included columella, root cap, and root border cell. This root border cell was seen before [42, 43], but was firstly described in this study by snRNA-seq.

**Fig. 2:**
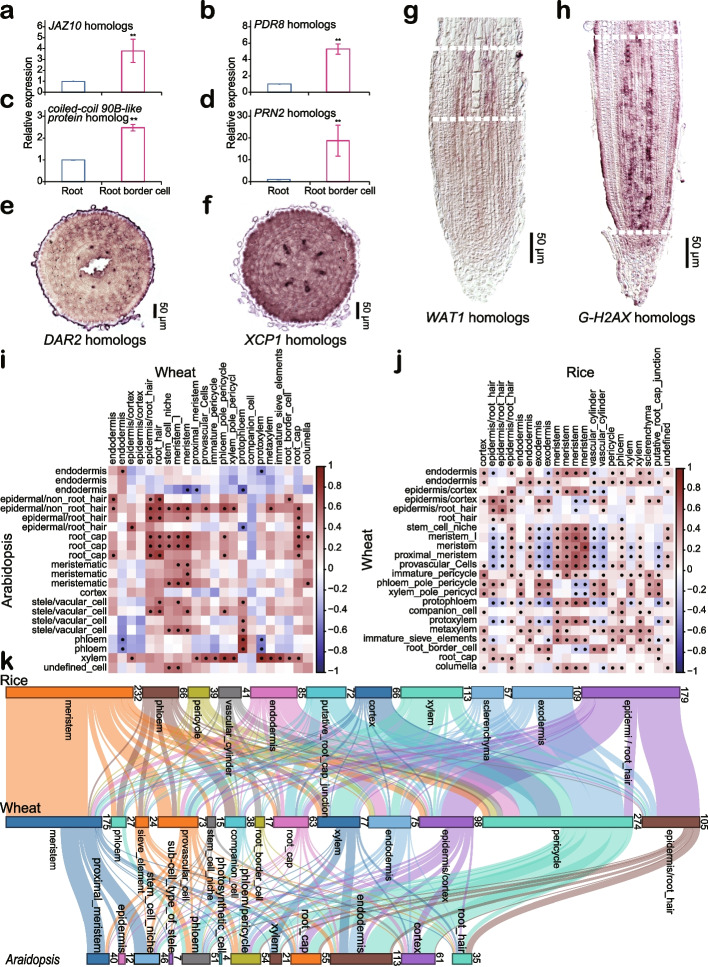
Validation of cluster-specific marker genes and comparison of root cell clusters of rice, *Arabidopsis* and wheat. **a–d** Relative expression of defense-related genes between root tips of radicles and root border cells. Means are calculated based on three independent biological replicates. Expression of *Actin7* was used as the endogenous control. *JAZ10* is the wheat homolog of *JASMONATE-ZIM-DOMAIN PROTEIN 10*;*PDR8* that represents the homologs of *PLEIOTROPIC DRUG RESISTANCE 8*; *PRN2 *that represent the homologs of *PIRIN2*. Asterisks indicate significant difference between the root tips of radicles and root border cells in Student’s* t* test (** *p*-value < 0.01). **e–h** RNA in situ hybridization assays to confirm the expression pattern of marker genes in root tips. *DAR2* homologs are the marker genes of companion cell (**e**); *XCP1* homologs are the marker genes of protoxylem (**f**). *WAT1* homologs are the marker genes of provascular cells (cluster 5) (**g**); *G-H2AX* homologs are the marker genes of proximal meristem (**h**). The zones labeled by two dashed white lines are the corresponding regions for provascular cells and proximal meristem, respectively. **i** Pairwise correlations of *Arabidopsis* (left) and wheat (top) root cell clusters. Dots indicate statistically significant correlations. **j** Pairwise correlations of rice (top) and wheat (left) root cell clusters. Dots indicate statistically significant correlations. **k** Sankey diagram showing that wheat shares a high degree of similarities in epidermal cell (root hair and no root hair), meristem, phloem (phloem, companion cell and sieve element), and xylem with rice and *Arabidopsis*. The number of overlapped orthologous genes for each cell type is given on the right of each cluster

We performed real-time quantitative PCR (qPCR) and RNA in situ hybridization assay to verify the expression of cluster-specific marker genes in root tips. Since root border cell typically detach from the root cap [42, 43], we collected root border cells from wheat root tips (Additional file 1: Fig. S5) (see “Methods”) and quantified the expression of homologs of *JASMONATE-ZIM-DOMAIN PROTEIN 10* (*JAZ10*), *PLEIOTROPIC DRUG RESISTANCE 8* (*PDR8*), and *PIRIN2* (*PRN2*). These genes were identified as marker genes in root border cells (cluster 19) (Additional file 2: Table S2). Indeed, their expression in root border cells was 2–18 times higher than that in the whole root tips (Fig. 2a–d). The *DA1-RELATED PROTEIN2* (*DAR2*) homologs were shown to be marker genes of companion cells (cluster 21) and were detected specific and radially oriented, dotted signal by RNA in situ hybridization (Fig. 2e). Moreover, the homologs of *XYLEM CYSTEINE PEPTIDASE 1* (*XCP1*) were identified as the marker genes of cluster 13. It showed quite similar radial but spatially distinct expression pattern in vasculature, which is characteristic of protoxylem (Fig. 2f). The homologs of *Arabidopsis WALLS ARE THIN 1 (WAT1)* were the marker genes of provascular cells (cluster 5). They were specifically expressed in the vasculature located at the starting point of elongation zone (Fig. 2g). The *WAT1* gene participates in the polar auxin transport and homeostasis of intracellular auxin metabolism, as well as cell elongation and secondary cell wall deposition [44–46], which is expressed in vascular cambium and xylem precursor cells in tomato [47]. Considering the molecular function and expressed pattern of *WAT1*, the result of RNA in situ hybridization assay further confirmed the provascular identity of cluster 5. The wheat homologs of *GAMMA HISTONE VARIANT H2AX* (*G-H2AX*) were identified as marker genes of cluster 3 and were expressed mainly in proximal meristem and primary meristem above the quiescent center (QC) (Fig. 2h). G-H2AX plays important roles in the DNA double-strand breaks (DSBs) sensing by recruiting DNA repair proteins and other factors at DSB sites to form the G-H2AX repair foci [48–53]. G-H2AX has been reported to regulate the rapid division rate of root meristem [50, 51]. The above results suggested the reliability of the cell type annotation.

We further performed pairwise comparisons of root cell clusters identified by the scRNA-seq/snRNA-seq data in *Arabidopsis*, rice and wheat (Fig. 2i–k) [21, 22]. The marker genes for clusters of endodermis, epidermis (root hair and no root hair), meristem, phloem (phloem, companion cell, and sieve element), and xylem showed a relatively high similarity in rice and wheat (Fig. 2j). The Sankey diagram showed significantly higher percentage of overlapped orthologous genes for meristem, phloem, and xylem between species (Fig. 2k). These results may suggest a conserved regulation network for meristem, phloem, and xylem development in plant.

### Differentiation trajectories of epidermal cells and ground tissue in wheat root

**Fig. 3:**
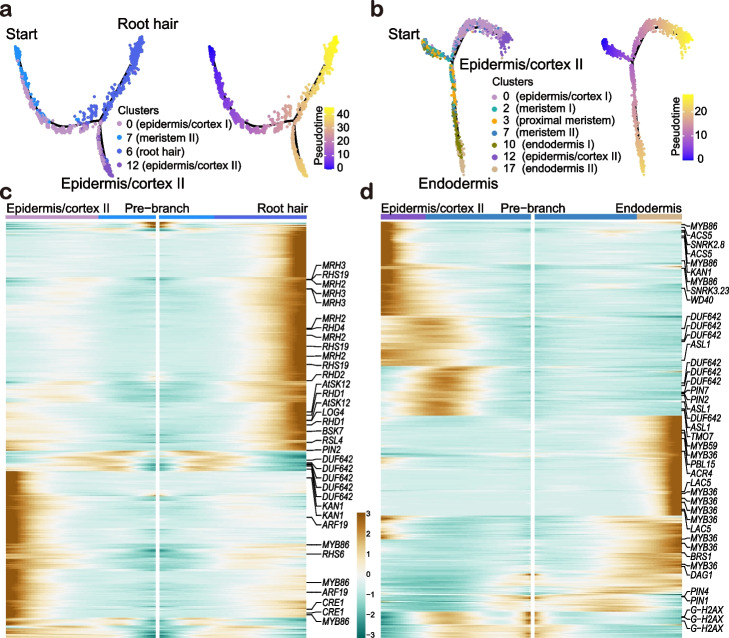
Differentiation trajectories of root hair, epidermis/cortex, and endodermis. **a,b** Differentiated trajectories of root hair and epidermis/cortex (**a**), epidermis/cortex and endodermis (**b**). Colors of dots are corresponding to cell clusters. The corresponding pseudo-time trajectory was shown on the right. **c,d** Branch heatmaps showing the expression of regulatory genes over the pseudo-time differentiation trajectories for root hair and epidermis/cortex (**c**), epidermis/cortex and endodermis (**d**). The pre-branch point is the beginning of pseudo-time. The annotated homologs of representative branch-dependent genes were shown on the right side of the branch heatmaps. Color bar indicates the relative expression level

Root hairs are essential for nutrient acquisition and uptake in plants. We constructed the pseudo-time developmental trajectory for epidermis/cortex and root hair. The results showed that the cortex/epidermis cells were initiated from the same meristematic cell cluster (cluster 7). The meristematic cells (cluster 7) were firstly differentiated into epidermis/cortex I (cluster 0) and subsequently, a subset of epidermis/cortex I (cluster 0) differentiated into epidermis/cortex II (cluster 12). Meanwhile, another subset of epidermis/cortex I (cluster 0) differentiated into root hairs (cluster 6) (Fig. 3a and Additional file 3). In order to get a deeper view on epidermis/cortex and endodermis development, we constructed pseudo-time developmental trajectories with clusters 2, 3, 7, 0, 12, 10, and 17. The cell clusters in charge of endodermis identity (clusters 10 and 17) were more related with cluster 3 (proximal meristem) than with cluster 7 (Fig. 3b). Recently, the cluster annotation in maize root also indicates the separated initials for endodermis and cortex although they have not pointed this out [18]. In conclusion, the observation in wheat is different from the results that the endodermis and cortex are differentiated from the same cells in *Arabidopsis* [20, 54–56].

To identify regulators controlling differentiation of root hair, epidermis/cortex, and endodermis, we identified the branch-dependent genes that are significantly enriched in pseudo-time trajectories. The homologs of *ROOT HAIR DEFECTIVE 1* (*RHD1*), *RHD2*, *RHD4*, *MORPHOGENESIS OF ROOT HAIR 2* (*MRH2*), *MRH3*, *ROOT HAIR SPECIFIC 19* (*RHS19*), and *ROOT HAIR DEFECTIVE 6-LIKE 4* (*RSL4*) contribute to root hair (cluster 6) development. On the other hand, the high expression of *KANADI 1* (*KAN1*), *AUXIN RESPONSE FACTOR 19* (*ARF19*), *CYTOKININ RESPONSE 1* (*CRE1*), and *MYB DOMAIN PROTEIN 86* (*MYB86*) homologous genes in the epidermis/cortex II branch emphasized their roles in epidermis/cortex II (cluster 12) fate determination (Fig. 3c, d, Additional file 2: Table S4 and Table S5). Most of the above genes showed relative specific expression in either epidermis/cortex II or root hair (Additional file 1: Fig. S6). High expression of root hair-related homologs of *RHD1*, *RHD2*, *RHD4*, and *RSL4* in cluster 6 indicated their conserved roles during root hair development in wheat and *Arabidopsis* [57–61]. In the trajectory for epidermis/cortex and endodermis differentiation, the homologous genes of *DOF AFFECTING GERMINATION 1* (*DAG1*), *BRI1 SUPPRESSOR 1* (*BRS1*), *MYB36*, *MYB59*, *LACCASE 5* (*LAC5*), *PBS1-LIKE 15* (*PBL15*), etc. were shown to direct endodermal cell differentiation (Fig. 3d and Additional file 2: Table S5). *MYB36* is a downstream target of SHR and regulates casparian strip formation in root endodermis in *Arabidopsis* [62–65]. This again supports a possible regulatory role of MYB36 for endodermis development in wheat.

### Subgenome asymmetric gene transcription in single-cell resolution

**Fig. 4:**
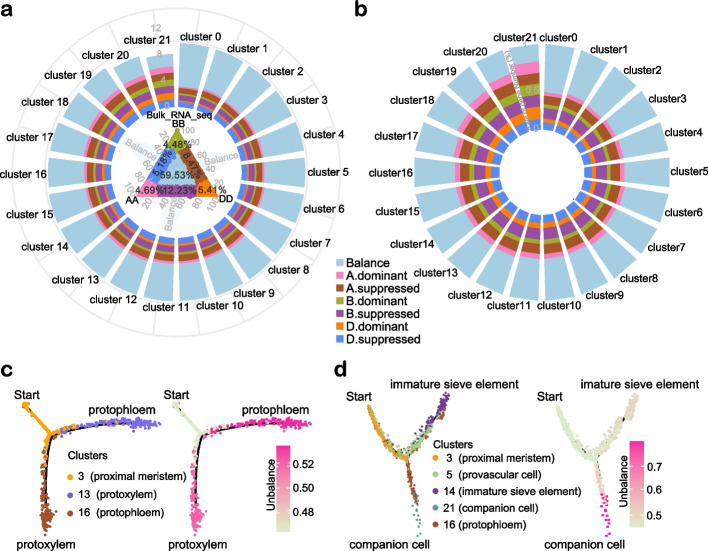
Cell-type-dependent asymmetric gene expression. **a** Balanced homoeologs in bulk RNA-seq show various unbalance expression patterns in different cell clusters defined by snRNA-seq data. The central ternary plot shows expression pattern of detected genes in bulk RNA-seq. The surrounding stacked histogram shows the cluster-specific expression pattern of balanced homoeologs defined by the bulk RNA-seq data. **b** Cell-type-specific asymmetric gene expression. The stacked histogram shows the relative proportion of genes with a certain biased expression pattern in the cluster. A. dominant represent the expression of genes located in A subgenome is higher than their homoeologs from B and D subgenomes; A. suppressed represent the genes located in A subgenome express at a lower level than their homoeologs from B and D subgenomes. Balance represent the homoeologs from A, B, and D subgenomes expressed at a similar level. **c,d** Ratios of asymmetrically expressed genes along with the differentiated pseudo-time trajectories for protoxylem and protophloem (**c**), companion cells and sieve element (**d**). Left panel is the pseudo-time trajectory. Right panel shows the ratios of asymmetrically expressed gene of the corresponding trajectory. Cells marked with “start” are the initial of cell differentiation trajectory, two ends of the branches are the differentiated cell types

It has been reported that around 30% of the wheat homoeolog triads (genes that have copies on A, B, and D genome) are expressed in an unbalanced manner [3]. However, this result was obtained from tissues that consist of multiple types of cells and represents the average gene expression from bulk cells. In order to obtain a high-resolution of asymmetric gene transcription atlas in polyploid wheat, we analyzed our snRNA-seq data and compared it with bulk RNA-seq from the same tissue. In general, the bulk RNA-seq data was highly correlated with the snRNA-seq data (Additional file 1: Fig. S2b). There were ~ 40% homoeolog triads that showed biased expression, while the other ~ 60% was balanced in the bulk RNA-seq data from AK58 roots (Fig. 4a and Additional file 2: Table S6). Interestingly, when the homoeologs with balanced expression pattern in the bulk RNA-seq were further analyzed in each root cell cluster identified by snRNA-seq, the expression of 31.7 to 76.1% genes, depending on cell clusters, were not balanced anymore. Particularly in cluster 21, only 23.9% of genes were expressed in a balanced manner (Fig. 4a and Additional file 5: Table S7). All these data strongly supported that the levels of asymmetric gene expression measured at single-cell resolution are highly heterogeneous across development. This has not been shown based on transcriptomics at bulk level in polyploid species.

The percentage of the homoeologs with unbalanced expression pattern varied between 40 and 55% in clusters 0–17, while this number increased to 60–80% in clusters 18–21 (Fig. 4b and Additional file 6: Table S8). These results indicate that homoeologs from A, B, and D subgenome tend to be more equally contributing to the development of vasculature, ground tissue and meristem than the stem cell niche, columella, root border cells, and companion cells. Interestingly, the proportion of suppressed genes in A, B, and D homoeolog triads was higher than the proportion of dominant ones in clusters 0–17 (A suppressed genes / A dominant genes = 1.76–2.4; B suppressed genes / B dominant genes = 2.22–3.0; D suppressed genes / D dominant genes = 1.4–1.95), but in cells from cluster 18–21, the ratios of A, B, and D dominant genes in homoeolog triads increased significantly (A suppressed genes / A dominant genes = 0.92–1.78; B suppressed genes / B dominant genes = 1.0–1.88; D suppressed genes / D dominant genes = 0.85–1.45) (Fig. 4b and Additional file 6: Table S8). GO analysis showed that increased asymmetric genes in cluster 19–21 mainly fall into the terms of endosome, *trans*-Golgi network, nucleoplasm, and cell division (Additional file 1: Fig. S7 and Additional file 2: Table S9). We studied asymmetric expression pattern of marker genes and their homoeologs (Additional file 1: Fig. S8 and Additional file 2: Table S10). We found that less than 45% of marker genes showed an unbalanced expression pattern in the stem cell niche, meristem, most vasculature, epidermis/cortex, and some transitional cell types in differentiation, while the well differentiated cell types, such as root border cells, columella, and companion cells (clusters 19–21) exhibited more than 50% marker genes with unbalanced expression pattern (Additional file 1: Fig. S8 and Additional file 2: Table S10).

We then studied the asymmetric expression of genes along the cell differentiation trajectory (Fig. 4c–d). For protophloem and protoxylem differentiation, their pseudo-ancestor cells (cluster 3) possessed only 46.5% of genes with unbalanced expression but this number increased to 52 and 53.4% for protophloem and protoxylem, respectively (Fig. 4c). As in the case of companion cell and immature sieve element cell, the proportion of genes with asymmetric expression pattern increased from 46.5 to 79.6% and to 51.3%, respectively (Fig. 4d). These results indicate that asymmetric gene expression among subgenomes may be an underlying driver of cell differentiation.

### Cell-type-specific regulatory network and key regulators for the root hair differentiation in wheat

**Fig. 5:**
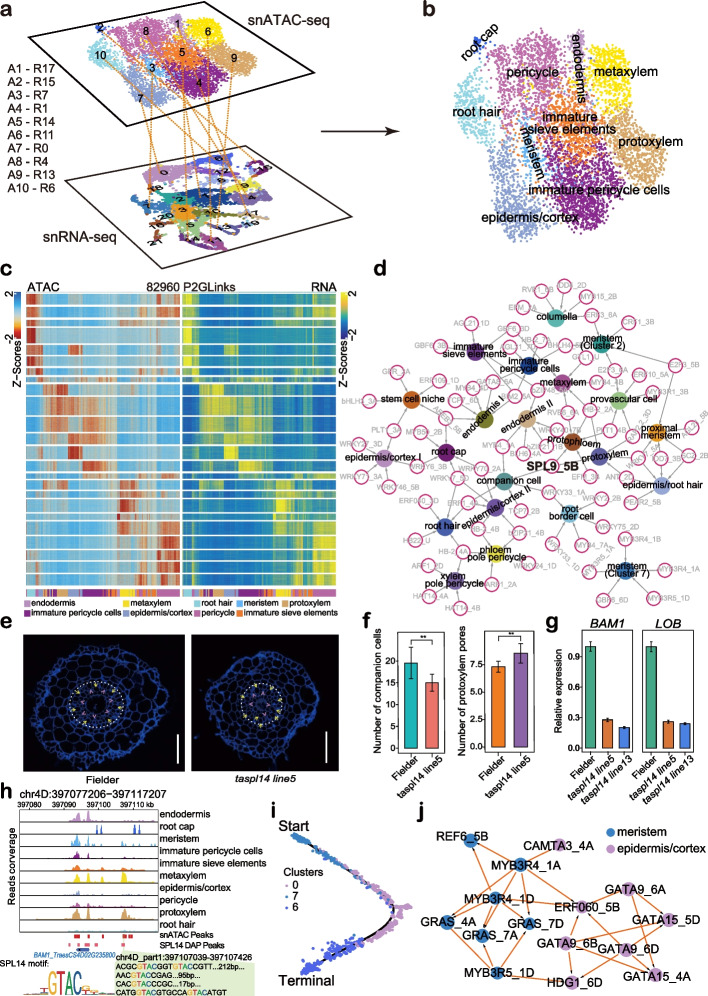
Cell-type-specific regulatory networks and key regulators for root hair differentiation. **a** Mapping the information of chromatin openness revealed by snATAC-seq data into the cell clusters classified by snRNA-seq. **b** UMAP visualization of 10 cell clusters were annotated by both snRNA-seq and snATAC-seq for wheat root. Each dot represents a single cell. **c** High correlation between chromatin accessibility and expression of marker genes for each corresponding clusters. Left part is the open chromatin scores calculated based on snATAC-seq, Right part is the expression level calculated based on snRNA-seq. **d** Top5 representative TF regulons for each cluster identified by SCENIC4. The abbreviated names of TF regulons were followed with the chromosome name to indicate their location in subgenomes. **e** Root transections of *taspl14* knock-out line and wild type. The protoxylem pores and companion cells were marked with yellow arrow and pink arrow head, respectively. Bar is 100 μm. **f** *taspl14* knock-out line showed reduced companion cells and increased protoxylem. **g** *BAM1* and *LOB* were downregulated in root of *taspl14* knock-out lines. **h** The accessible chromatin regions (ACRs) of *BAM1* homolog (*TraesCS4D02G235800*). **i** Differentiated trajectories of root hair. Colors of dots are corresponding to cell clusters. Start indicates the initiation of the pseudo-time trajectory. Terminal indicates the end of the pseudo-time trajectory. **j** Trajectory network for root hair differentiation identified the key regulators for cell identity transition

To explore cell-type-specific regulatory networks, we associated gene transcription with dynamic accessible chromatin regions (ACRs) at a genome-wide scale. Firstly, we mapped open chromatin regions at single-cell level in the wheat root by snATAC-seq. We clustered the cells based on the openness of chromatin identified by snATAC-seq and correlated them with snRNA-seq (Fig. 5a, b). In total, 10 clusters were well annotated and correlated. In each cell type, the high gene scores calculated based on ATAC-seq peak were highly indicative of gene expression, showing a high correlation between the snRNA-seq and snATAC-seq data (Fig. 5c). Cluster-specific marker genes presented in snRNA-seq UMAP plots also showed cluster-specific pattern in snATAC-seq UMAP plots, which indicated high relevance between the snRNA-seq and snATAC-seq dataset (Additional file 1: Fig. S9). The cluster representative *cis*-motifs were also identified by using snATAC-seq data (Additional file 1: Fig. S10).

To systematically reveal cell-type-specific regulatory networks, SCENIC4 was applied for TF-centered regulon identification based on co-expression and motif enrichment [66, 67]. In total, we identified 185 TF regulons across 22 root cell types (Additional file 1: Fig. S11a and Additional file 2: Table S11) and representative TF-based regulon modules, including *ETHYLENE RESPONSE FACTOR 043* (*ERF043*, *TraesCS3D02G141200*), *PLT1* (*TraesCS4B02G235900*), *MYB3R4* (*TraesCS1B02G147800*), and *Basic Helix-Loop-Helix130* (*BHLH-130*, *TraesCS5D02G272800*), were shown to be highly cell-type specific (Additional file 1: Fig. S11a and S11b). The top5 representative TFs for each cell cluster are shown in Fig. 5d (Additional file 1: Fig. S11, S12 and Additional file 2: Table S12). *MYB3R4* and *MYB3R5* homologs were active regulons in cluster 7 with meristematic activity. MYB3R4 has been demonstrated to promote cell division, activated by cytokinin in meristem [68]. In the cell types of pericycle (clusters 1, 4, and 9), homologs of the *AUXIN RESPONSE FACTOR 1* (*ARF1*) and *HOMEOBOX DOMAIN-2* (*HB-2*) presented high activities, which is consistent with their role of local morphogenetic trigger of auxin to specify the lateral root formation in pericycle [69–74]. *HB-2* has been demonstrated to regulate lateral root formation in *Arabidopsis* [70, 71]. In addition, *AGAMOUS-Like21* (*AGL21*) was also an active regulon in pericycle (cluster 1). *AGL21* positively regulates accumulation of auxin in lateral root primordia and promotes the development of lateral root [75, 76]. The overlapping regulons among clusters 1, 4, and 9 further supported their pericycle cell identity. *MYB4* led active regulons in endodermis (clusters 10 and 17), metaxylem (cluster 11), root border cells (cluster 19), and companion cells (cluster 21), corresponding to a role of *Arabidopsis* homologs in lignification [77, 78]. In line with the possible function in defense regulation [79–82], *WRKY33* had been isolated as a key regulator in root border cells (cluster 19). Interestingly, the *SQUAMOSA PROMOTER BINDING PROTEIN-LIKE 9* (*SPL9*) homolog *TaSPL14* was identified to be key regulator in several vasculature cell types, including protoxylem (cluster 13), protophloem (cluster 16), and companion cells (cluster 21) (Fig. 5d). SPLs has been reported to activate the *MADS box* genes at shoot apex to promote flowering in *Arabidopsis* [83, 84], but their specific function in root vascular development largely remains unknown. To verify the specific regulatory functions of SPLs in root vasculature, we explored the vascular phenotypes of two *taspl14* knock-out lines generated by CRISPR/Cas9 [85]. The roots of the *taspl14* knock-out line showed reduced number of companion cells and increased protoxylem pores in the root transection compared to wild type (Fig. 5e, f). We also identified homologs of *BARELY ANY MERISTEM1* (*BAM1*) and *LATERAL ORGAN BOUNDARIES* (*LOB*) as predicted target genes of *TaSPL14* and their expression were downregulated in root of the *taspl*14 knock-out line (Fig. 5g). Interestingly, BAM1 has been demonstrated to repress the protophloem differentiation and to be required for normal root xylem patterning [86, 87], which is consistent with the activity of *TaSPL14* in both of protoxylem (cluster 13) and protophloem (cluster 16). The chromatin located at upstream of *BAM1* homolog (chr4D_part1:397,107,039–397,107,426 and *TraesCS4D02G235800*) was only specifically accessible in protoxylem, metaxylem, and meristem and the binding motifs of TaSPL14 were enriched in this region. Interestingly, we also detected the binding of TaSPL14 to this region by analyzing a published DAP-seq data [88] (Fig. 5h). Hence, we proposed that the TaSPL14-BAM1 module acts as an important role for vasculature development in wheat root. Moreover, we also investigated the expression of marker genes, including homologs of *DAR2*, *LSD ONE LIKE 3* (*LOL3*), *ANNEXIN 5* (*ANN5*), *ALTERED PHLOEM DEVELOPMENT* (*APL*), *VND-INTERACTING 2* (*VNI2*), *TARGET OF MONOPTEROS 5* (*TMO5*), *XCP1*, and *VASCULAR RELATED NAC-DOMAIN PROTEIN 1* (*VND1*), most of which has been demonstrated to play important roles in protoxylem (cluster 13), protophloem (cluster 16), and companion cells (cluster 21) [89–94]. Four out of these eight genes showed statistically significant change in *taspl14* knock-out line, including the homologs of *LSD ONE LIKE 3* (*LOL3*) in companion cells, the homologs of *ALTERED PHLOEM DEVELOPMENT* (*APL*) [89] in protophloem, and the homologs of *XCP1* [91] and *VASCULAR RELATED NAC-DOMAIN PROTEIN 1* (*VND1*) [90] in protoxylem (Additional file 1: Fig. S13).

We were wondering whether it is possible to identify regulators that control cell fate change during cell differentiation. We took root hair development as an example to answer the question. Based on pseudo-time developmental trajectory, root hair cell originates from a specific meristem cell population (cluster 7), which was then differentiated into epidermis/cortex I for root hair initiation (Fig. 5i). To explore key regulators to determining cell fate decisions, we firstly identified the differentially expressed genes along the pseudo-time trajectory. The changed expression of these genes was regarded as the reason for the cell fate determination. Using the regulatory relationship identified by SCENIC and TF imprints revealed by ATAC-seq, TFs that drive the change of gene expression during the switch from one cell type to the other were identified. By this approach, we identified major TFs that promote differentiation from the meristematic cell to epidermis/cortex I, as well as TFs that are responsible for root hair differentiation from epidermis/cortex I. In detail, homologs of *MYB3R4*, *REF6*, *GRAS*, and *MYB3R5* were strongly associated with changing meristematic cells (cluster 7) into epidermis/cortex I while *HOMEODOMAIN GLABROUS1* (*HDG1*), *GATAs*, *ERF060*, and *CAMTA3* were TFs that were active in the epidermis/cortex I and implicated in root hair development (Fig. 5j). *HDG1* belongs to the same class IV homeodomain-Leucine zipper gene family together with *GLABRA2* (*GL2*), *ANTHOCYANINLESS2* (*ANL2*), *FLOW-ERING WAGENINGEN* (*FWA*), *ARABIDOPSIS THALIANA MERISTEM LAYER1* (*ATML1*), *PROTODERMAL FACTOR2* (*PDF2*), and *HDG1*, which had been demonstrated to be involved in epidermal cell differentiation. *Arabidopsis GL2* was negative regulator for root hair development [95–99]. These results showed that cell-type-specific networks and key regulators can be uncovered and the genes identified here could be important targets to understand root development and root trait.

## Discussion

The specific morphology of the root greatly contributes to plant adaptation to the environment, such as semi-dry land or paddy field. Our snRNA-seq results show that the wheat root is a complex organ consisting of more than 20 distinct cell types. Many homologs of regulators of *Arabidopsis* root cell identity are also specifically expressed in corresponding wheat root cell clusters (Additional file 1: Fig. S14 and Additional file 2: Table S2). This indicated potentially conserved regulatory mechanisms in root cell differentiation comparing wheat and *Arabidopsis*. For example, the homologs of *TMO5* were identified as marker genes for protoxylem [94, 100–102], and homologs of *DAR2* were marker genes for companion cells in wheat root (Additional file 1: Fig. S14 and Additional file 2: Table S2) [92]. For the ground tissue, we proposed the conserved function of MYB36 to regulate endodermis development between wheat and *Arabidopsis* (Fig. 3d) [62–65].

Besides anatomical characterization, the development of the root is not well characterized in most grass species. In this study, we analyzed the detailed differentiation trajectories of ground tissue in wheat. Trajectories leading to ground tissue formation appear to be diverged between wheat and *Arabidopsis*. Although we cannot distinguish cortex and epidermis, they are quite likely differentiated from the same group of meristematic cells. Moreover, endodermis and epidermis/cortex were found to have the different origin in the wheat root (Fig. 3b). In contrast, the cortex and endodermis appear to have a common initial in *Arabidopsis* root [20, 54–56]. Interestingly, the pseudo-time trajectory in maize root also indicated the common initiation of epidermis and cortex [18]. This seems to be a common phenomenon in most monocots, in which the cortex and epidermis originates from the same initial and asymmetrically divides to produce the multilayer cortex [31, 54, 103–106]. Pairwise comparison of the scRNA-seq data of rice and *Arabidopsis* suggested that rice endodermis related with some *Arabidopsis* vascular cells, but not the *Arabidopsis* endodermis [22]. This is consistent with the observation that wheat root endodermis differentiated from the proximal meristem, which mainly developed into vascular cells. The different initials of endodermis and cortex were also presented in maize root [18]. Allopolyploidy causes a “genome shock” and alters the expression pattern of many genes [107, 108]. The activities of homoeologs from different subgenomes need to be coordinated to reach transcriptional homeostasis. It is widely accepted that exploring the transcriptional coordination mechanisms of homoeologs facilitates crop improvement. It was reported that about 30% of homoeolog triads showed unbalanced expression [3]. Our results demonstrate that the balanced expressed genes in bulk RNA-seq showed unbalanced expression pattern at single-cell resolution. More interestingly, lower ratio of unbalanced expressed genes existed in vasculature, meristem, ground tissue, and epidermal tissue, but stem cell niche, columella, root border cells, and companion cells showed higher unbalanced expression pattern (Fig. 4b). The proportion of suppressed genes in clusters 0–17 was consistent with previous studies of genes’ inactivation that contributes to the expression bias in polyploid wheat [3, 10, 109]. But this suppression tended to be interrupted in clusters 18–21. The cell division-related genes showed balanced expression in divided and differentiated potential cells, while they showed either no existence or asymmetric expression in the well differentiated mature cells, as well as stem cell niche. The asymmetric ratios increased along with the differentiated trajectory of vasculature which indicated that the asymmetric gene expression is an underlying drive for cell specification. These results also suggest that specific coordination or asymmetry expression among homoeologs may act as important roles in root cell identity maintenance, which can avoid the coexistence of some conflict genes or other unexpected negative effects.

The marker gene annotation and differentiated trajectories provided insight for root cell organization and alternately variation of important genes along with the differentiated trajectories. Moreover, the identification of cell-type-specific regulons provided further supplementary information for better understanding of cell type identities. Here, we also integrated the information of gene expression and active open chromatin, to construct the regulatory network along with the differentiated trajectory of root hairs. This trajectory network identified key TFs inducing identity conversion between adjacent cell types along with the trajectory.

## Conclusions

In this study, we constructed the single-cell atlas for wheat root. We surveyed asymmetric transcription at single-cell resolution and revealed that the asymmetry pattern of homoeologs is cell-type dependent in wheat root. In addition, cell differentiation trajectory suggested that epidermis/cortex and endodermis originated from different meristems in wheat root, which differs from the situation in *Arabidopsis*, but is similar to the situation in maize and rice. We also combined the active ACRs of high variable genes along with pseudo-time trajectory of root hair development and constructed the trajectory-specific regulation network. This trajectory network highlighted the upstream TF (transcriptional factors) regulation in root hair differentiation. Taken together, we described wheat root organization and particularly highlighted the asymmetry heterogeneity of gene expression in single-cell resolution in polyploidy species, which may contribute to the precision breeding for polyploidy species toward traits of root.

## Methods

### Plant materials and growth conditions

The wheat variety of Aikang 58 (AK58) was used for root tip collection and snRNA-seq studies [110, 111]. The AK58 seeds were sterilized with 75% ethanol for 3 min and followed with 4% NaClO_2_ for 5 min. After washing the seeds with sterile water for 5 times, the seeds are kept in sterile water for 12 h. Then, the seeds were transferred on the 1/2 Murashige and Skoog (1/2 MS) plates with vertical placement at 22℃ under the condition of 16 h light and 8 h dark per day. The root tip of AK58 grown 3 days was collected for bulk RNA-seq. *taspl*14 knock-out lines 5 and 13, generated by the CRISPR/Cas9, were kindly provided by Dr. Yingyin Yao [85].

### Nuclei isolation for snRNA-seq and snATAC-seq

The 0.5-cm root tips were harvested and frozen by liquid nitrogen for subsequent snRNA-seq and snATAC-seq experiments. The nuclei isolated procedures are as follows: chop the root tips of AK58 in 0.4 M D buffer (0.4 M D-sorbitol, 20 mM 2-morpholinoethanesulphonic acid, 20 mM KCl, 10 mM MgCl_2_, 0.2% Triton X100, 3 mM Dithiothreitol, and 1% bovine albumin) with RNase inhibitor in it on pre-chilled plates. The homogenates were resuspended with 400 μl 0.4 M DW buffer (0.4 M D-sorbitol, 20 mM 2-morpholinoethanesulphonic acid, 20 mM KCl, 10 mM MgCl_2_, 3 mM Dithiothreitol, and 1% bovine albumin). The plate was shaken for 1 min on ice. The lysate was transferred into a 35-μm strainer and the follow through collected using a 1.5-ml RNase-free Eppendorf tube. Then, 400 μl 0.4 M D buffer was added and the liquid transferred into a 35-μm strainer and the follow through collected using a 1.5-ml RNase-free Eppendorf tube. Four microliters DAPI (2.5 mg/ml) was added to the solution and stained for 5 min in dark. The nuclei was sorted by BD Aria III, and 300 μl DW buffer was used with RNase inhibitor (Ribo Lock RI 400 U/ml; SuPERase In RI 200 U/ml) to collect the sorted nuclei. The tube was horizontally centrifuged at 500* g*, at 4℃ for 6 min. The supernatant was transferred into a new RNase-free Eppendorf tube with 20 μl supernatant retained at the bottom of the tube. Five hundred microliters DW buffer was added to resuspend the precipitation and centrifuged at 500 g, at 4℃, for 6 min. The supernatant was transferred into a new RNase-free Eppendorf tube, with 20 μl supernatant retained at the bottom of the tube. One hundred microliters PBS buffer (for snRNA-seq) or 40 μl Nuclei buffer (for snATAC-seq) was added to resuspend the precipitation. The concentration of nuclei was counted by the BodBogeautomatic cell counter. Then we loaded approximately 15,000 nuclei (for snRNA-seq) and 10,000 transposed nuclei (for snATAC-seq) to Chromium Single Cell Instrument.

### snRNA-seq and snATAC-seq library construction and sequencing

The single-nuclei suspension was loaded into 10 × Genomics Chromium Single Cell Chip B for snRNA-seq. The generated single-nuclei GEMs by Chromium Controller were used to generate the snRNA-seq library according to the user guide of Chromium Single Cell 3’ Reagent Kits v3 (CG000183 Rev A). Then, the resulting DNA library was analyzed by Agilent 2100 Bioanalyzer. Libraries were sequenced on an Illumina NovaSeq 6000 paired-end sequencing run (2 × 150 bp).

For snATAC-seq, the tagmentation follows the protocol of 10 × Genomics Chromium Single Cell ATAC. Then, 10,000 tagmentated nuclei were loaded into 10 × Genomics Chromium Single Cell Chip E for snATAC-seq. The generated single-nuclei GEMs by Chromium Controller were used to generate the snATAC-seq library according to the user guide of Chromium Single Cell ATAC Reagent Kits (CG000168 Rev A). The resulting DNA library was analyzed by Agilent 2100 Bioanalyzer. Libraries were sequenced with Illumina NovaSeq 6000 in dual-index mode with 8 and 16 cycles for i7 and i5 index, respectively.

### Raw data pre-process for snRNA-seq and snATAC-seq

Cell Ranger 6.0.1 (10 × Genomics) were used to analyze the raw snRNA-seq dataset (CRA008788) [112] with the parameters of –expect-cells = 15,000 and –include-introns. The IWGSC RefSeq v1.0_parts pseudomolecule (161010_Chinese_Spring_v1.0_pseudomolecules_parts.fasta) [113] was used as reference genome. The gtf file was self-modified according to the annotation file of IWGSC v1.1 [114]. We used poly dT to capture the RNAs in each nucleus and we took the advantage of our nucleus based method to include introns and 3′ UTR, 5′ UTR to count the abundance of RNAs. Only unique mapped reads with MAPQ value of 255 was used to generate a UMI count matrix where each row contains a single gene and each column represents a cell. The chloroplast (NC_002762) and mitochondrial (NC_036024) genomes were downloaded from NCBI database and analyzed separately to assess the sequencing quality [115, 116].

The raw snATAC-seq reads (CRA008788) [112] were processed and aligned to the IWGSC RefSeq v1.0_parts pseudomolecule reference genome [113] using Cell Ranger ATAC (v2.0). The full-featured R package of ArchR (v1.0.1) was used to complete the subsequent analysis [117], including doublet removal, dimensionality reduction, cell clustering, gene score calculation, peak calling, and multiomic integration with snRNA-seq data (CRA008788) [112].

### Cell clustering, and annotation for snRNA-seq data

We applied Seurat package (v.4.0.4) [118] for the further analysis of the gene-cell matrices. Genes that expressed in fewer than 3 cells were discarded, and cells with featured RNA number lower than 200 or higher than 100,000 were deduced. Then, the function of “NormalizeData” was performed using LogNormalize method and scale factor of 10,000. The functions of “FindVariableFeatures” (vst method, 2000 features) and “ScaleData” were performed to detect variable genes and scale data, respectively. The functions of “RunPCA” (100 principal components) and “JackStraw” were applied for reducing dimensions. The “FindNeighbors” with dims set to 1:49 and “FindClusters” with resolution of 0.8 were used to construct the SNN graph and to cluster the cells. Finally, the function “RunTSNE” and “RunUMAP” were performed to visualize the result with non-linear dimensional reduction algorithms.

In the “FindAllMarkers” function of Seurat, we set logfc.threshold = 0.25 and min.pct = 0.25 to find the cluster-enriched genes. We classified and annotated the clusters according to the known functions and expression pattern of genes enriched in each cluster (Additional file 2: Table S2 and Additional file 4). The expression of the representative marker genes was visualized by “DotPlot” function of Seurat. The 3D UMAP plot was visualized by R with Plotly package (v4.9.4.1).

### Correlation analysis of snRNA-seq and bulk RNA-seq

The bulk RNA-seq datasets (CRA008788) [112] of AK58 roots with two biological repeats were used for correlation analysis with snRNA-seq dataset (CRA008788) [112]. For bulk RNA-seq, we quantified the expression of each transcript using TPM value via kallisto (v0.44.0) [119]. Further, the transcript TPM matrix was aggregated to gene TPM matrix by Sleuth (v0.30.0) [120]. For snRNA-seq, we calculated the average of gene expression using “geneAverageExpression” function of Seurat. The Spearman correlation coefficient was calculated and visualized using R (v4.0.0).

### Long noncoding RNA analysis

The sequence of wheat long noncoding RNA (*Triticum aestivum* (Ensembl Plants 51).fasta) was downloaded from a Wiki-database of plant lncRNAs (v2.0) GreeNC (http://greenc.sequentiabiotech.com/wiki2/Main_Page) [121]. The expression number of the long noncoding RNAs in each cell was calculated according to the average expression level. The Shannon entropy specificity index was calculated to identify the cell specifically expressed long noncoding RNAs via Tspex (https://tspex.lge.ibi.unicamp.br/). Long noncoding RNAs with highest Shannon entropy specificity index in each cell cluster were visualized using modified VlnPlot function of Seurat. The long noncoding RNAs which were identified as markers in each cell clusters were visualized via pheatmap package (v1.0.12) in R.

### Pairwise comparisons of root cell clusters in wheat, rice, and Arabidopsis

The average expression of orthologous marker genes in each cell cluster from wheat and the published scRNA-seq datasets in *Arabidopsis* (PRJNA517021, GSE123013, and GSE123818) [122–124] and rice (PRJNA706435 and PRJNA706099) [125, 126] were used to perform the cross-species pairwise cluster comparisons as described [21, 22, 127]. The orthologous genes among wheat, rice, and *Arabidopsis* were downloaded from the Triticeae-Gene Tribe (http://wheat.cau.edu.cn/TGT/index.html) (Additional file 2: Table S13). The number of overlapped orthologous genes for each cluster was visualized using sankeyD3 (v0.3.2) package in R.

### Motif enrichment analysis

After the groups of cells are defined and pseudo-bulk replicates created, we generated reproducible peaks for each cell cluster via ArchR (v1.0.1) using MACS2 as peak caller [117]. Then, the getfasta function of BEDTools v2.27 [128] was used to extract the sequence of each peak. The peak sequences in each cell cluster were submitted into the CentriMO (https://meme-suite.org/meme/tools/centrimo) online analysis toolkit for motif enrichment analysis, respectively. The “anywhere” mode was selected to perform local motif enrichment, and the motif database used was CIS-BP 2.0.

### Pseudo-time analysis

To explore the molecular mechanism of cell differentiation and cell fate determination, we performed pseudo-time analysis using Monocle v.2.20.0 [129]. Firstly, we defined the marker genes with *p* adjust value lower than 0.05 as the developmental progress specific genes. Secondly, we set up the parameter ‘‘max_components” as two and method as “DDR Tree’’ to reduce the dimensionality of the data into two components. In the lower dimensional space, the “orderCells” function was performed to order the cells in pseudo-time according to the transcriptome correlation. Further, we performed the “plot_cell_trajectory” function to visualize the cell trajectory. To designate the priori “start point” in the trajectory, the “orderCells” function was performed again with appointing the ‘‘root_state’’. The branch-dependent genes were analyzed by “BEAM” function. Then, we performed “plot_genes_branched_heatmap” to demonstrate the bifurcation of gene expression along two branches.

### Root border cell collection for real-time quantitative PCR (qPCR)

The AK58 seeds were germinated and grown for 3 days on 1/2 MS plates vertically at 22℃ under 16 h light and 8 h dark per day. Then, the root was washed with 1/2 MS liquid medium for 24 h. We collected the liquid that contained the detached root border cells. The liquid was centrifuged at 200* g* for 10 min at 4℃. After the supernatant was discarded, the detached root border cells were obtained (Additional file 1: Fig. S5).

### Real-time quantitative PCR (qPCR) analysis

Total RNA was extracted from the root tips and root border cells with the TRIzol reagent (Invitrogen, production No. 15596026). Then, the cDNA was synthesized with the One-Step gDNA Removal and cDNA Synthesis SuperMix (TransGen Biotech, production No. AE311–02). The real-time quantitative PCR was performed on CFX connect (Bio-Rad) with 2 × Universal SYBR Green Fast qPCR Mix (ABclonal, production No. RK21203). Primers for qPCR are listed in Additional file 2: Table S14.

### In situ RNA hybridization assays

The forward and the reverse primers containing T7 promoter sequence were used to amplify the coding region of a gene to prepare template. Primers are listed in Additional file 2: Table S14. The template and T7 RNA polymerase (Roche, Cat No./ID: 10,881,767,001) were incubated for the transcription of digoxigenin-labeled RNA probe. We performed the in situ RNA hybridization by following the protocol as previously described by Yang et al. [130]. The 0.5-cm wheat root tips were harvested and fixed with formaldehyde. The sections of paraffin-embedded samples with 10–12-μm thickness were placed on coated glass slides. The slides with sample sections were dewaxed with Histoclear and digested with Proteinase K (Roche, Cat No./ID: 03,115,828,001). Then, gradient ethanol series (up to the 100% ethanol) were used to dehydrate the slides. The prepared slides with sample sections were hybridized with the digoxigenin-labeled RNA probe. After incubation with anti-digoxigenin-AP Fab fragments (Roche, Cat No./ID: 11,093,274,910), the slides were washed and the signal was detected with the solution of NBT/BCIP (Roche, Cat No./ID: 11,681,451,001).

### Subgenome asymmetric expression analysis

The asymmetric expression analysis of bulk RNA-seq and snRNA-seq were based on the genes triads across the homoeologs of A, B, and D subgenomes. A total of 17,311 syntenic homoeolog triads for 51,933 genes were extracted from the Triticum_aestivum_V1_PGSB.homeologous_gene_pairs.txt file downloaded from the Wheat-URGI data repository [113] and used for further analysis. To standardize the relative expression of each homoeolog gene across the triad, we normalized the expression level so that the sum of the expression of the three genes in each triad is 1. After the Euclidean distance of each triad was calculated, we classified the triads into seven categories as previously defined, i.e., Balance, A.dominant, B.dominant, D.dominant, A.suppressed, B.suppressed, and D.suppressed [3]. The R package ggtern v3.3.5 was used to visualize the subgenome asymmetric expression of each triad.

### GO enrichment analysis

GO enrichment was performed using Triticeae-GeneTribe GOEnrichment toolkit (http://wheat.cau.edu.cn/TGT/), and the top 5 GO terms were visualized using R package ggplot2 v3.3.5.

### Paraffin section analysis

The 0.5-cm wheat root tips were harvested and fixed in formaldehyde-aceticacid-alcohol. Then, we dehydrated the root tips with series of gradient ethanol solution. Subsequently, gradient ratio of (xylene: ethanol, up to the 100% xylene) was used to transparentize the root tips. The samples were incubated in a xylene:paraffin solution at 42℃ for 24 h. The root tips were embedded in paraffin for 3 days. The sections of paraffin-embedded samples with 10-μm thickness were placed on coated glass slides. The slides with sample sections were dewaxed with Histoclear and rehydrated with gradient ethanol series (down to 30% ethanol). The images of root tips were taken using fluorescence microscope (Leica). The vasculature and transection area were measured with ImageJ software.

### Identification of cell-type-specific regulons

Python frame (pySCENIC) was applied to investigate the gene regulatory network and key regulators that characterize a particular cell type [67]. Briefly, the filtered gene-barcode count matrix was served as input to construct the TF-gene co-expression modules using GRNBoost2, followed by prediction of candidate regulons using the ctx command. Regulon activities were calculated in each cell using the AUCell command. The wheat TF motifs used to create the pre-requisite *cis*-target database were collected from the CIS-BP (https://jaspar.genereg.net/) and JASPAR (http://cisbp.ccbr.utoronto.ca/) website. The regulators of the top 5 regulons in each cell type were extracted and then visualized using Cytoscape v3.8.1 [131].

### TF regulatory network for root hair differentiation

To explore the key TF regulators controlling root hair developmental process, we firstly used the clusters 0, 6, and 7 to preform pseudo-time analysis and identify the variable genes along with the pseudo-time trajectory. Subsequently, we extracted the regulatory relationships in SCENIC-inferred regulons (genetic units comprised by a group of genes regulated by a key TF regulatory) that the expression of key regulator or their target genes changed along with the root hair development pseudo-time trajectory. Then, TFs imprinting analysis was performed to detect the upstream TF regulators of target genes whose expression changed along with developmental trajectories. In detail, the peak-gene pairs were detected from scATAC-seq dataset using the ArchR “addPeak2GeneLinks” function. Peaks obtained from peak-gene pairs and high-quality mapped reads generated from Cell Ranger were used to perform TF-footprint analysis and create the TF binding network using TOBIAS (https://github.com/loosolab/TOBIAS). The regulatory network extracted from cluster-specific regulons and inferred from TF imprinting analysis were integrated and visualized via Cytoscape v3.8.1 [131].

## Supplementary Information

**Additional file 1:** **Fig. S1.** Status of sorted nuclei before loading into 10× genomics chips. **Fig. S2. **Estimation of the data quality. **Fig. S3. **t-SNE visualization of 22 cell clusters annotated for wheat root tips. **Fig. S4. **Expression of long noncoding RNAs in each cluster. **Fig. S5.** Root border cell detached from root cap. **Fig. S6. **Genes specifically expressed in epidermis/cortex and root hair. **Fig. S7. **Top 10 GO categories for balanced and unbalanced genes, respectively. **Fig. S8. **Expression bias of cluster specific marker genes. **Fig. S9. **UMAP plots showing cluster specificity of common marker genes between the corresponding clusters of snRNA-seq and snATAC-seq. **Fig. S10. **Representative motifs for each cluster of snATAC-seq. **Fig. S11.** 185 TF regulons across 22 root cell types identified by SCENIC4. **Fig. S12. **Top5 representative TF for each cell cluster. **Fig. S13. **Relative expression of marker genes of companion cells, protophloem and protoxylem in *taspl14* line 5. **Fig. S14. **Homologs of conservative genes between *Arabidopsis* and wheat root specifically expressed in corresponding wheat root cell clusters.

**Additional file 2:** **Table S1.** The comparison of single-cell datasets generated from wheat and *Arabidopsis* root. **Table S2.** Top 100 of cluster-specific marker genes. **Table S3.** The expression of matrix of long noncoding RNAs for each cluster. **Table S4.** The expression of branch-dependent genes over the differentiated pseudo-time for epidermis/cortex and root hair. **Table S5.** The expression of branch-dependent genes over the differentiated pseudo-time for endodermis and epidermis/cortex. **Table S6.** Gene expression bias based on bulk RNA-seq of AK58 root. **Table S9.** GO enrichment of balance and unbanlance genes for each cluster. **Table S10.** Expression bias of marker genes in each cluster of AK58 root. **Table S11.** Hub genes of 185 regulons acrosss all clusters identified by SCENIC4 soft. **Table S12.** Hub genes of top5 regulons for each cluster analyzed by SCENIC4 soft. **Table S13.** The orthologous genes of wheat, rice and *Arabidopsis thaliana*. **Table S14.** Primers used for qPCR analysis and template synthesis for in situ RNA hybridization.

**Additional file 3.** 3D UMAP scatterplot.

**Additional file 4.** Supplementary Note.

**Additional file 5:** **Table S7.** Cluster specific expression bias of balanced genes in bulk RNA-seq of AK58 root.

**Additional file 6:** **Table S8.** Expression bias of expressed genes in each cluster of AK58 root.

**Additional file 7.** Review history.

## Data Availability

All the raw datasets of snRNA-seq (CRR602489), snATAC-seq (CRR602491), and bulk RNA-seq (CRR602490 and CRR602492) generated from this study have been deposited in the Genome Sequence Archive at the Beijing Institute of Genomics (BIG) Data Center, Chinese Academy of Sciences, under accession number CRA008788 (https://ngdc.cncb.ac.cn/gsa/browse/CRA008788) [112]. The IWGSC RefSeq v1.0_parts pseudomolecule reference genome (iwgsc_refseqv1.0_all_chromosomes.zip) was downloaded from IWGSC Data Repository hosted at URGI (https://urgi.versailles.inra.fr/download/iwgsc/IWGSC_RefSeq_Assemblies/v1.0/) [113]. The sequences of chloroplast and mitochondrial were downloaded from the nucleotide database in National Center for Biotechnology Information under the accession numbers of NC_002762 [115] and NC_036024 [116], respectively. The root single-cell RNA sequencing datasets in *Arabidopsis* were from the three published studies and downloaded from NCBI SRA or GEO (PRJNA517021, GSE123013, and GSE123818) [122–124]. The single-cell RNA sequencing datasets in rice was downloaded from NCBI SRA with the accession number of PRJNA706435 and PRJNA706099 [125, 126]. The annotation of wheat long noncoding RNA was downloaded from a Wiki-database of plant lncRNAs (v2.0) GreeNC (http://greenc.sequentiabiotech.com/wiki2/Main_Page) with species name *Triticum aestivum* (Ensembl Plants 51) [121]. The self-modified gtf file based on the annotation file of IWGSC v1.1 was deposited under GPL-3.0 license in Github (https://github.com/hcph/wheat_singlecellAtlas) [114] and in Zenodo (https://zenodo.org/record/7761965) [132]. There are no custom scripts and codes used other than those mentioned in the “Methods” section.
