# Supplementary material for: Asymmetric gene expression and cell-type-specific regulatory networks in the root of bread wheat revealed by single-cell multiomics analysis
Source: Genome Biol. 2023 Apr 4;24:65. doi: 10.1186/s13059-023-02908-x (PMC10074895; doi:10.1186/s13059-023-02908-x)
Supplement: Supplementary file 1 — Additional file 1: Fig. S1. Status of sorted nuclei before loading into 10× genomics chips. Fig. S2. Estimation of the data quality. Fig. S3. t-SNE visualization of 22 cell clusters annotated for wheat root tips. Fig. S4. Expression of long noncoding RNAs in each cluster. Fig. S5. Root border cell detached from root cap. Fig. S6. Genes specifically expressed in epidermis/cortex and root hair. Fig. S7. Top 10 GO categories for balanced and unbalanced genes, respectively. Fig. S8. Expression bias of cluster specific marker genes. Fig. S9. UMAP plots showing cluster specificity of common marker genes between the corresponding clusters of snRNA-seq and snATAC-seq. Fig. S10. Representative motifs for each cluster of snATAC-seq. Fig. S11. 185 TF regulons across 22 root cell types identified by SCENIC4. Fig. S12. Top5 representative TF for each cell cluster. Fig. S13. Relative expression of marker genes of companion cells, protophloem and protoxylem in taspl14 line 5. Fig. S14. Homologs of conservative genes between Arabidopsis and wheat root specifically expressed in corresponding wheat root cell clusters. [file 13059_2023_2908_MOESM1_ESM.pdf]

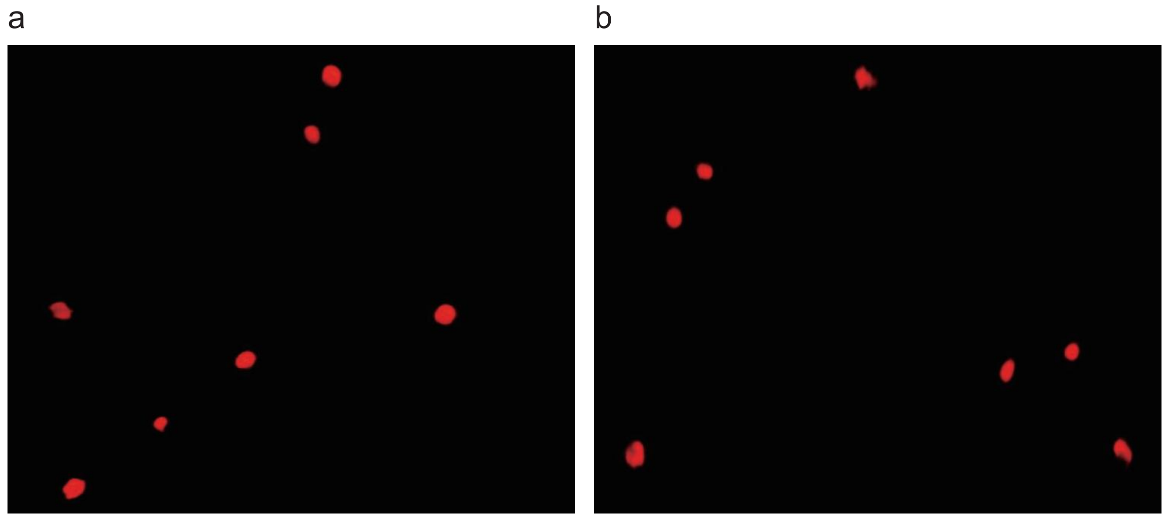

Fig. S1 Status of sorted nuclei before loading into 10× genomics chips. (a) Status of sorted nuclei for snRNA-seq. (b) Status of sorted nuclei for snATAC-seq.

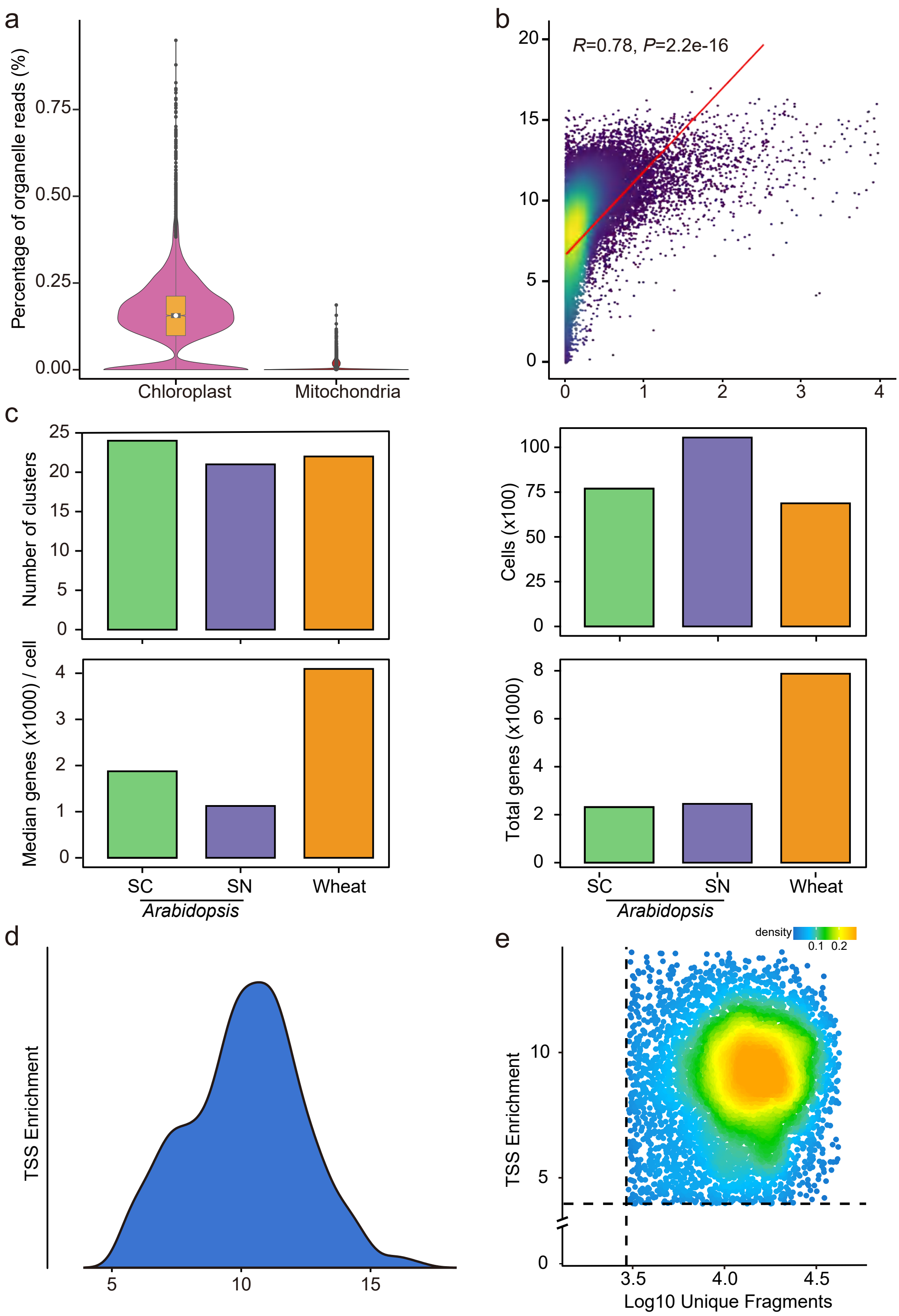

Fig. S2 Estimation of the data quality. (a) The percentage of organelle reads in the snRNA-seq dataset. (b) The bulk RNA-seq data is highly related with the snRNA-seq data. (c) The basic comparison of single nuclei or cell RNA sequencing in different species root. *Arabidopsis*_sc means the data generated from single cell RNA sequencing in *Arabidopsis*. *Arabidopsis*_sn means the data generated from single nuclei RNA sequencing in *Arabidopsis*. (d) The accessible regions enriched around the transcription start sites (TSS). (e) QC filtering plots showing the TSS enrichment covered unique nuclear fragments per cell. Dot colors represent the density in arbitrary units of points in the plot.

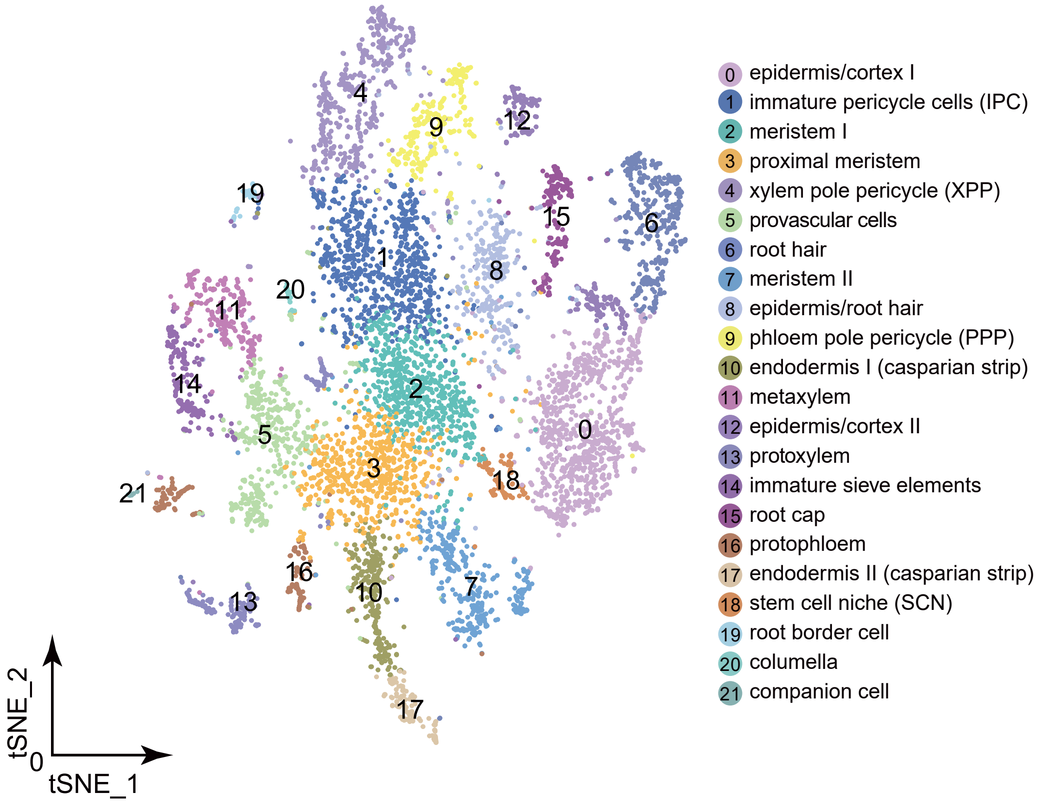

Fig. S3 *t*-SNE visualization of 22 cell clusters annotated for wheat root tips. Each dot represents a single cell.

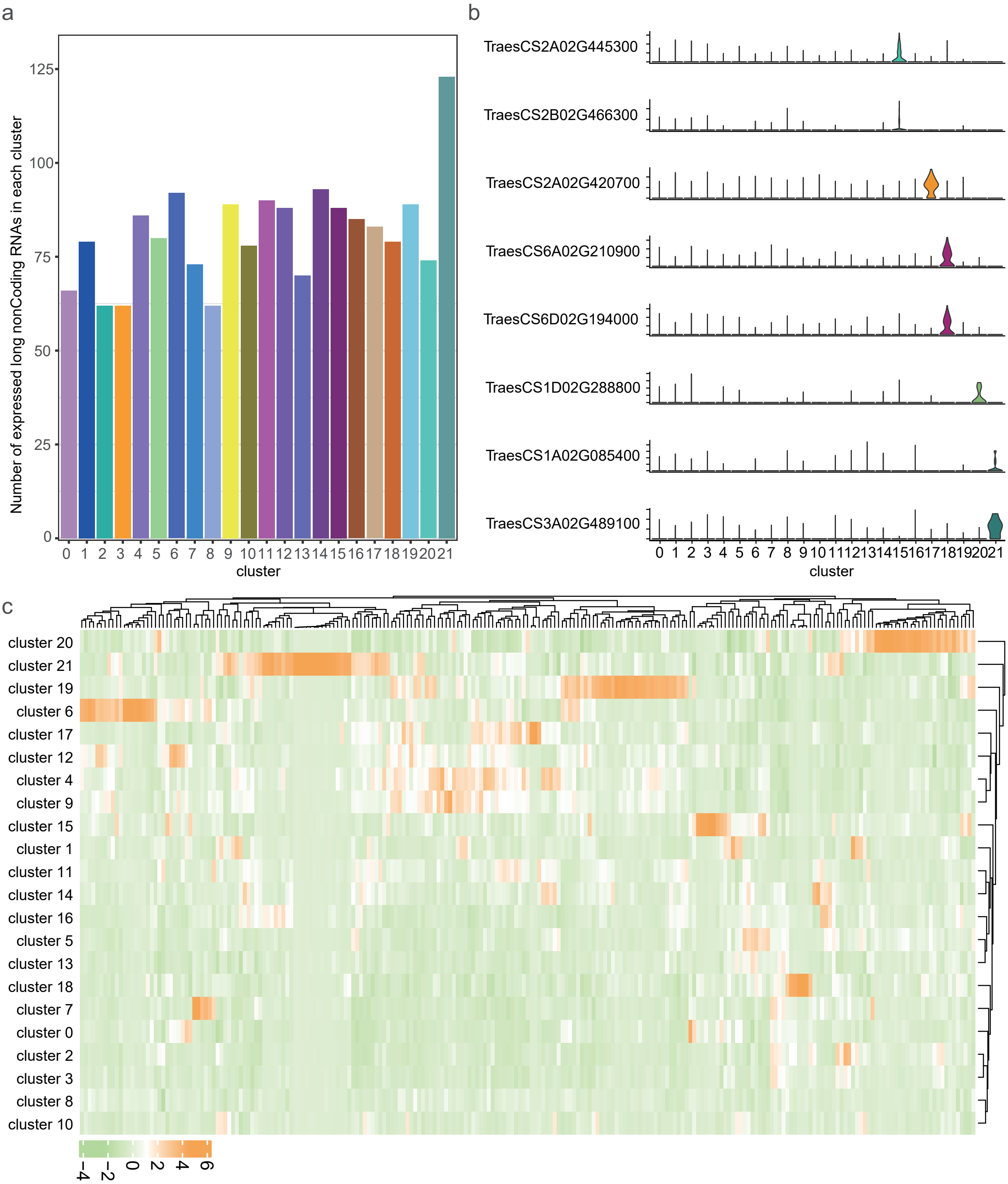

Fig. S4 Expression of long noncoding RNAs in each cluster. (a) Number of expressed long noncoding RNAs in each cluster. (b) Representative long noncoding RNAs of each cluster. (c) Heatmap showing the cluster enriched long noncoding RNAs in each cell clusters.

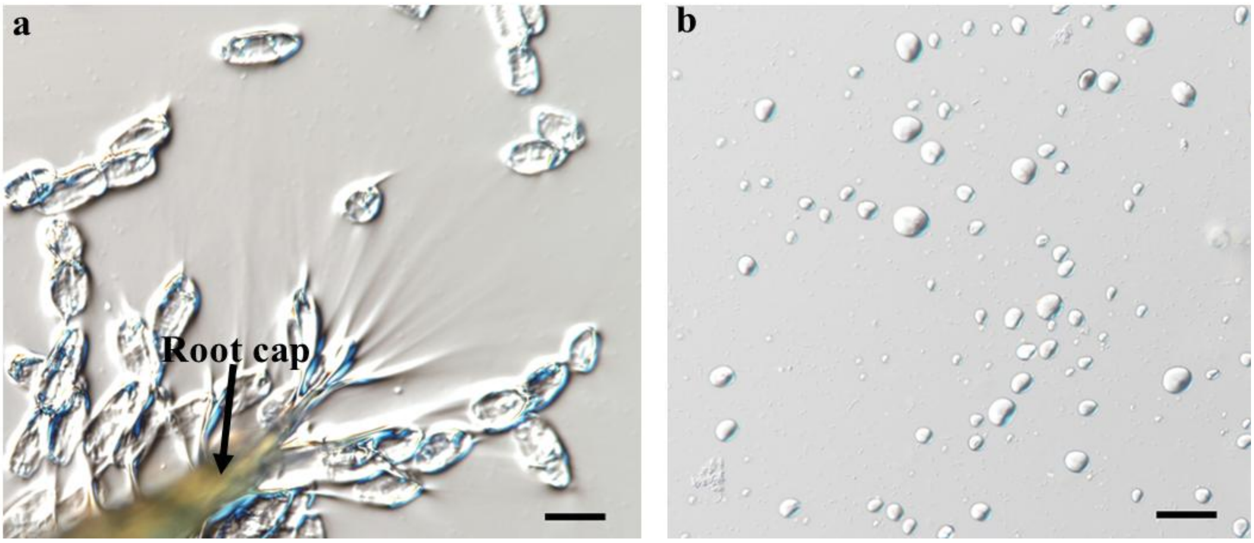

Fig. S5 Root border cell detached from root cap. (a) The root border cell detached from root cap on the glass slide. (b) The root border cell collected from 1/2 Murashige and Skoog liquid medium. Bar is 100 μm.

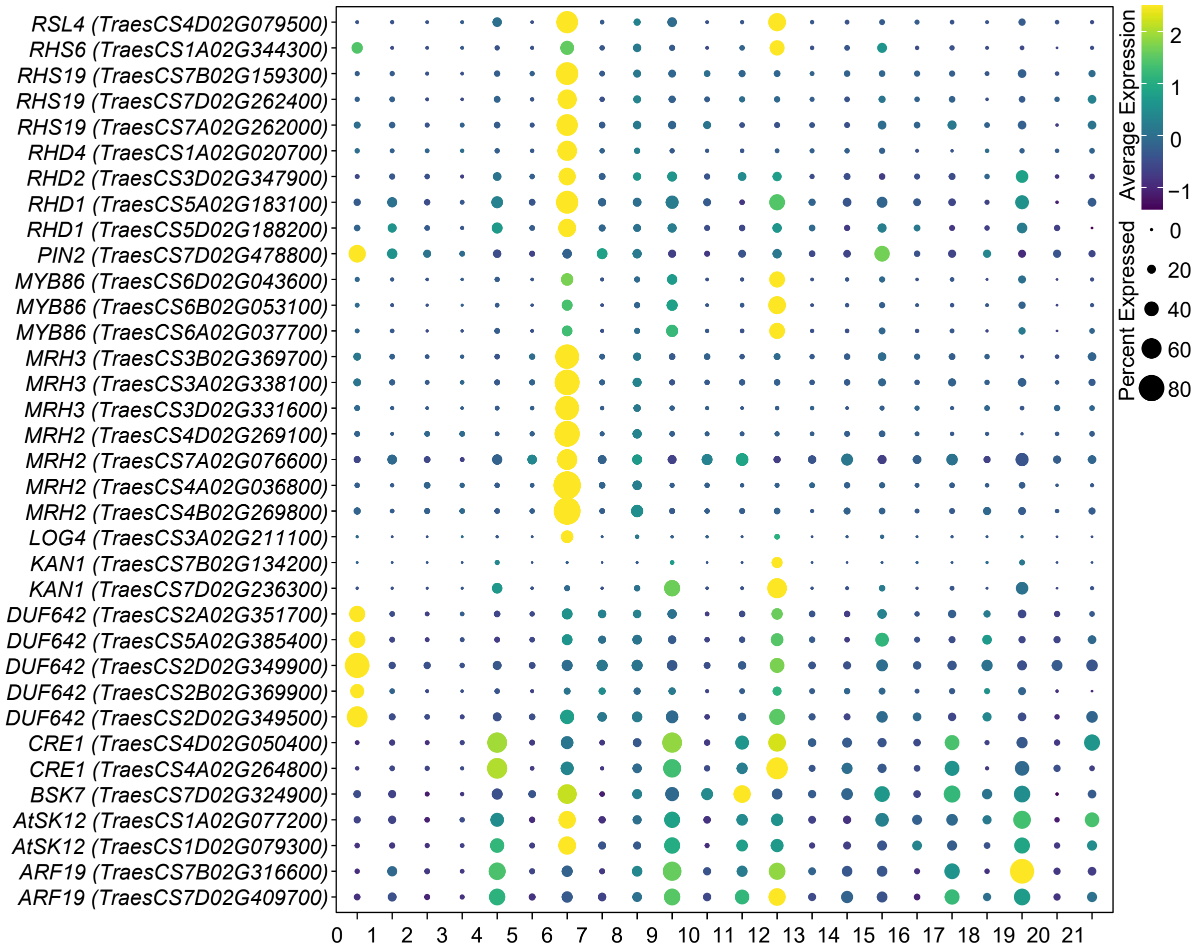

Fig. S6 Genes specifically expressed in epidermis/cortex and root hair.

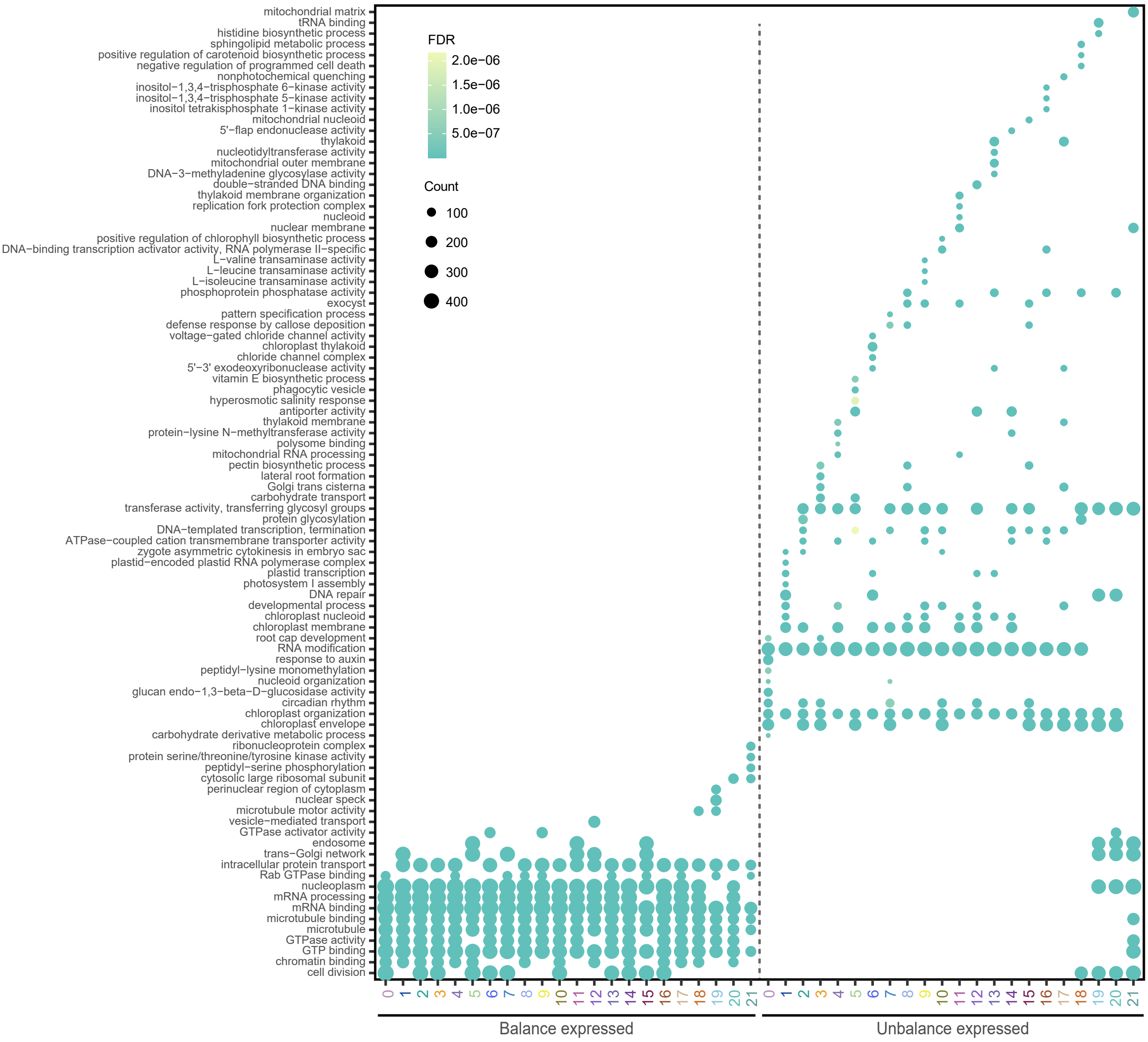

Fig. S7 Top 10 GO categories for balanced and unbalanced genes, respectively.

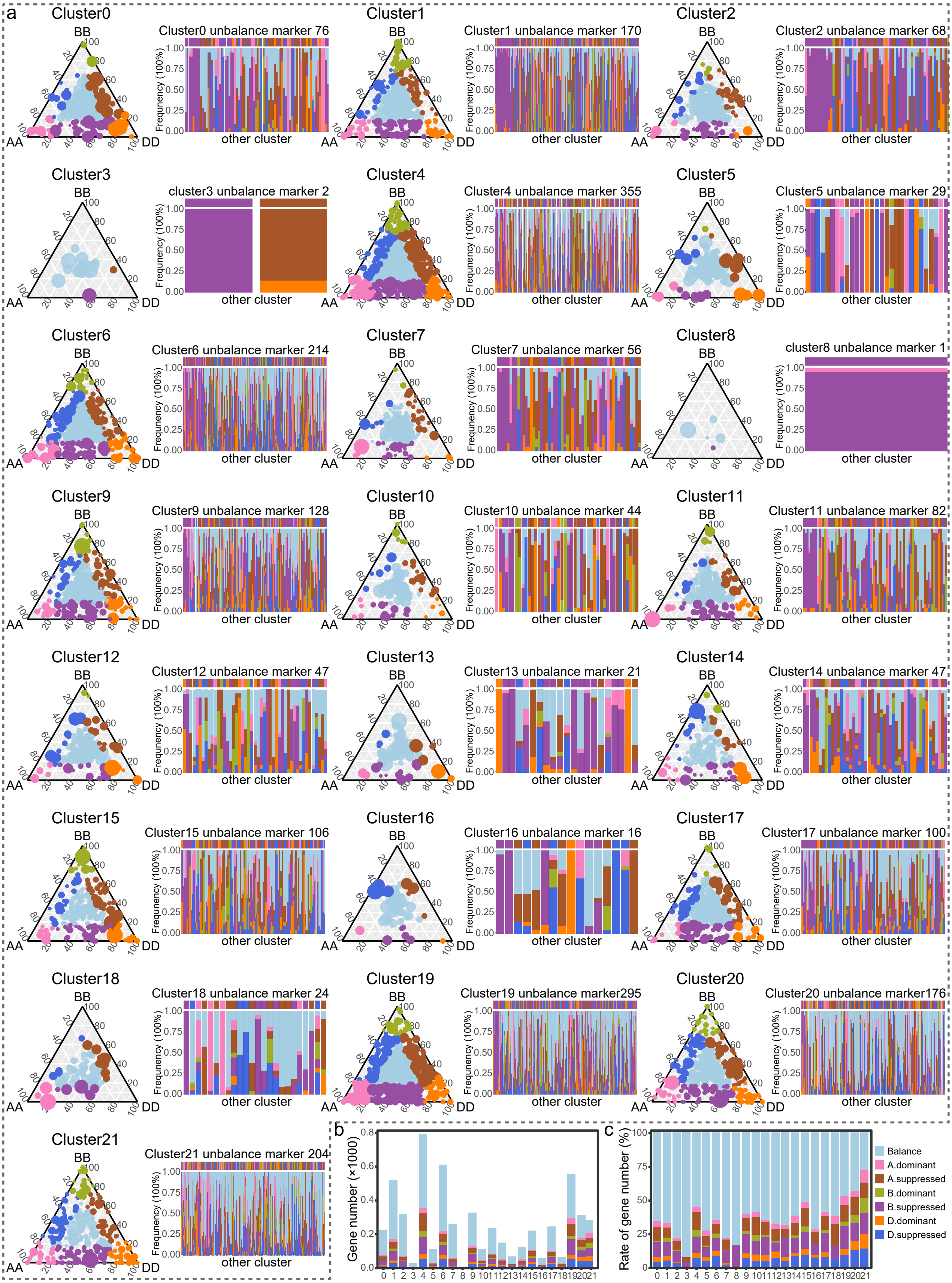

Fig. S8 Expression bias of cluster specific marker genes. (a) The expression bias of marker genes in 22 clusters. For each cluster, the left part is the ternary plot showing expression pattern of marker genes. The right part is the stacked histogram showing the expression pattern for each gene in other clusters. (b) The number of balanced and asymmetrically expressed genes among cluster specific marker genes. (c) The percentage of cluster specific marker genes with different expression bias. Different color represents the corresponding expression bias pattern in the ternary plots.

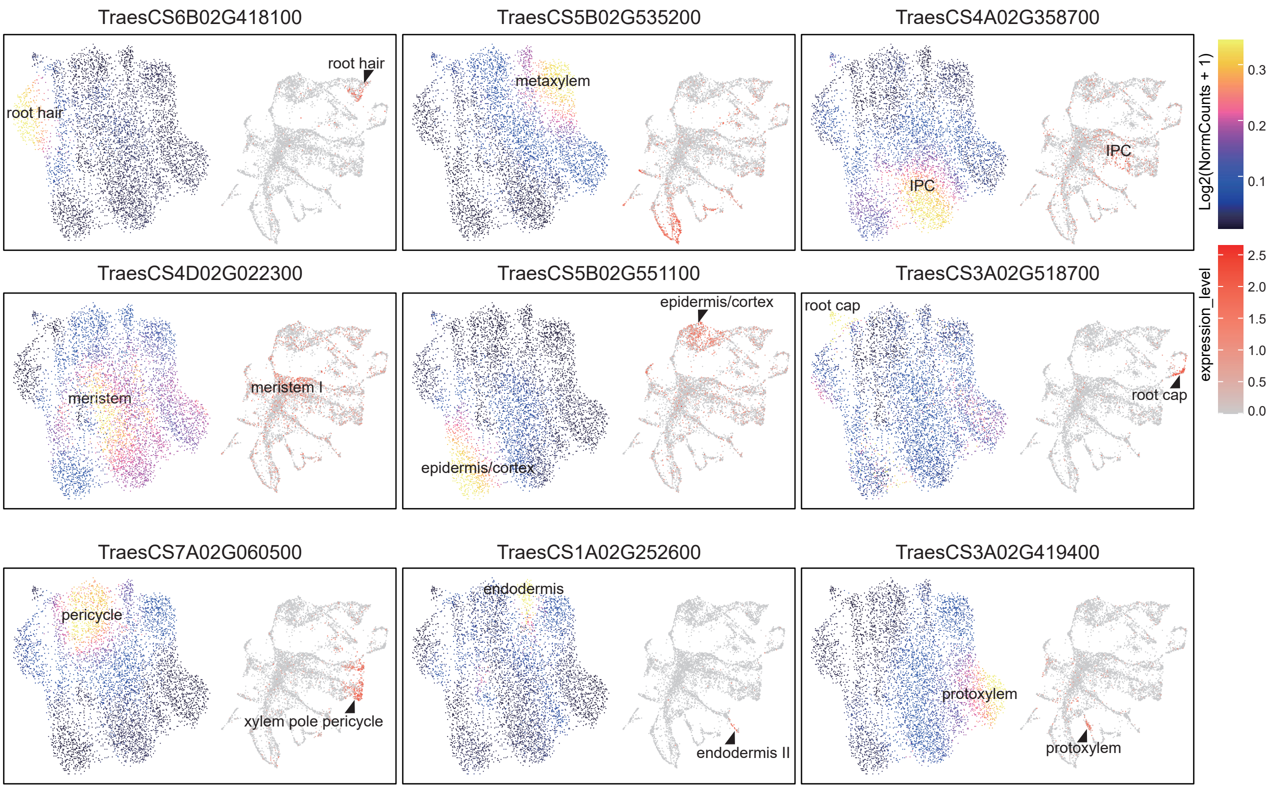

Fig. S9 UMAP plots showing cluster specificity of common marker genes between the corresponding clusters of snRNA-seq and snATAC-seq.

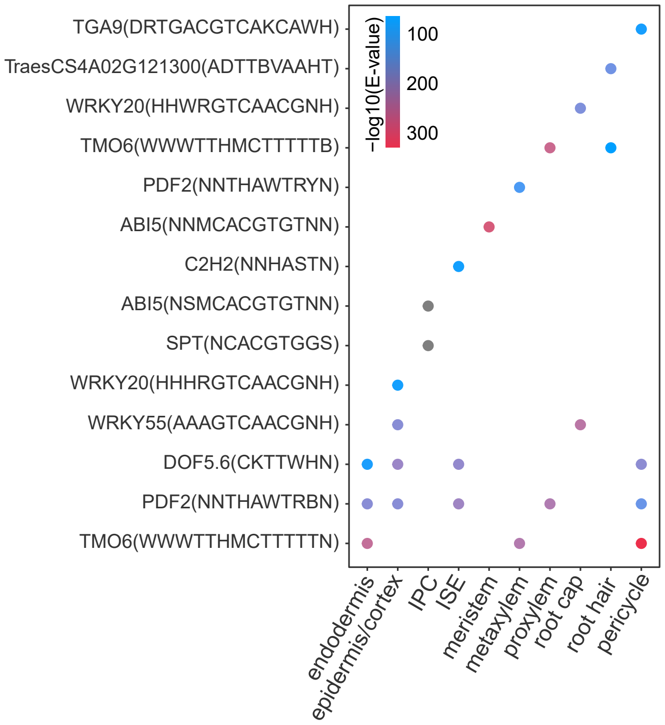

Fig. S10 Representative motifs for each cluster of snATAC-seq.

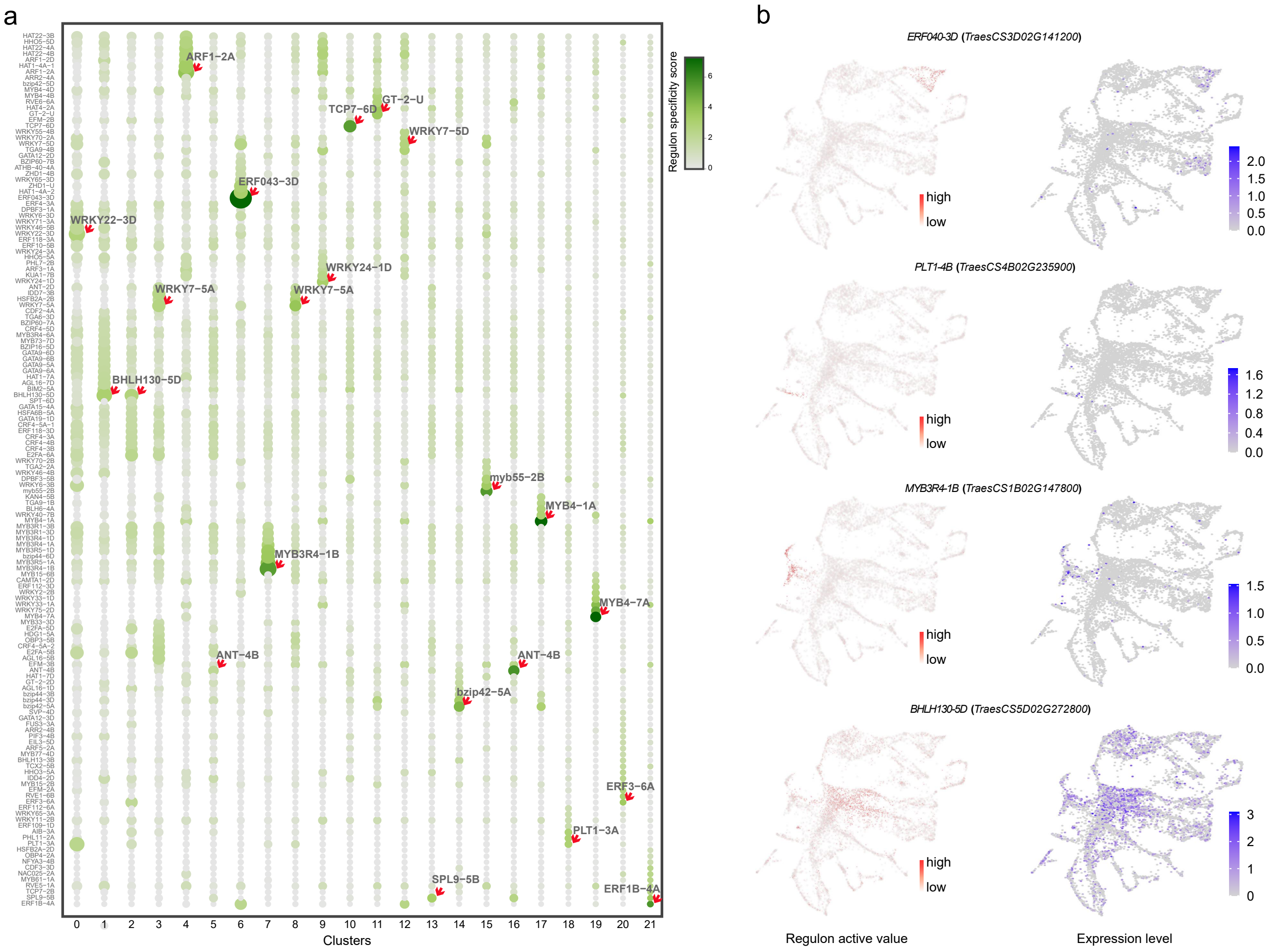

Fig. S11 185 TF regulons across 22 root cell types identified by SCENIC4. (a) 185 TF regulons and their cell type specificity score across 22 root cell types. (b) Expression level and activity of representative regulons in the UMAP plot.

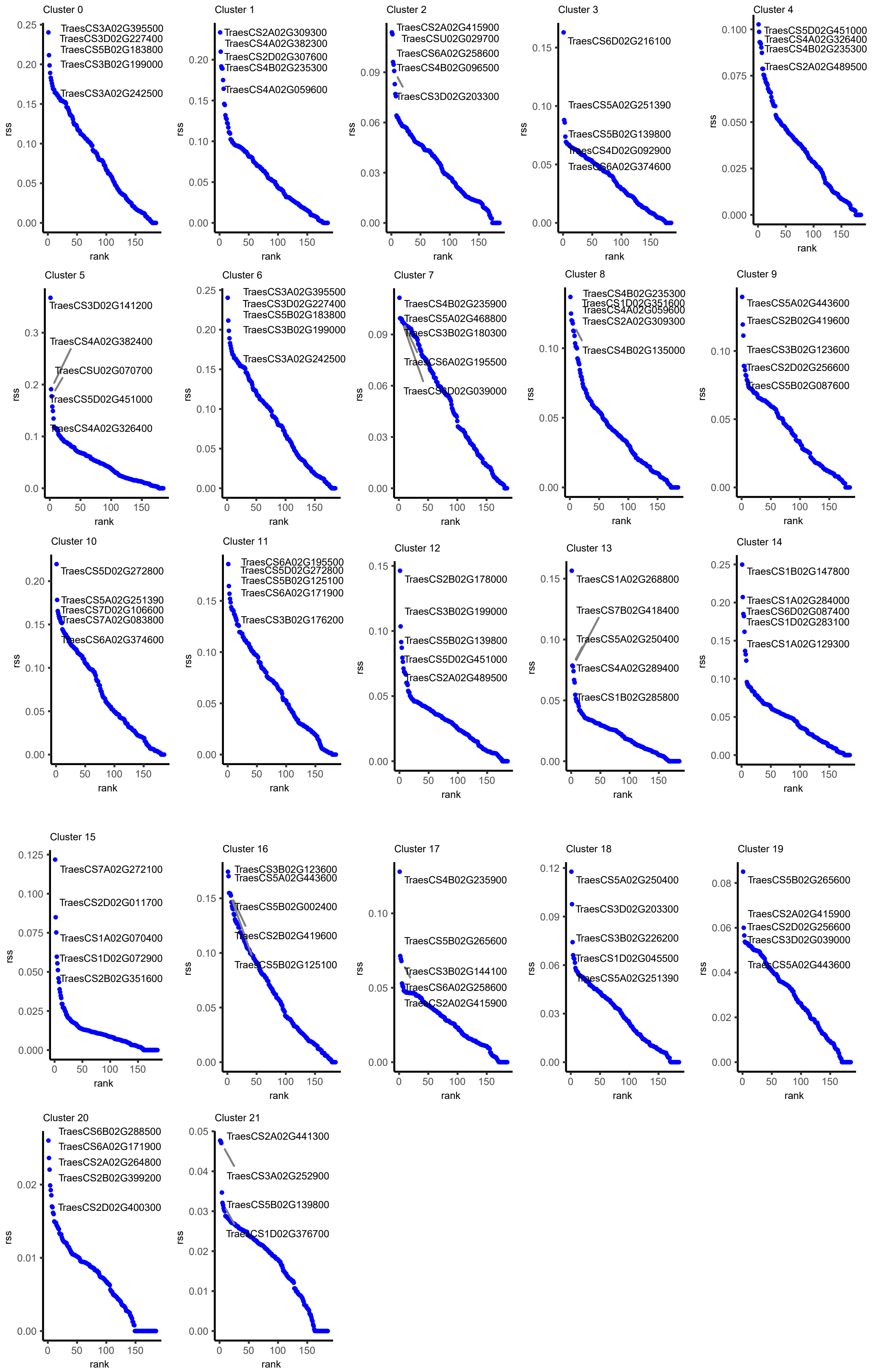

Fig. S12 Top5 representative TF for each cell cluster. The y axis indicates the regulon specificity score (rss); the x axis indicates the rank of regulons for each cluster.

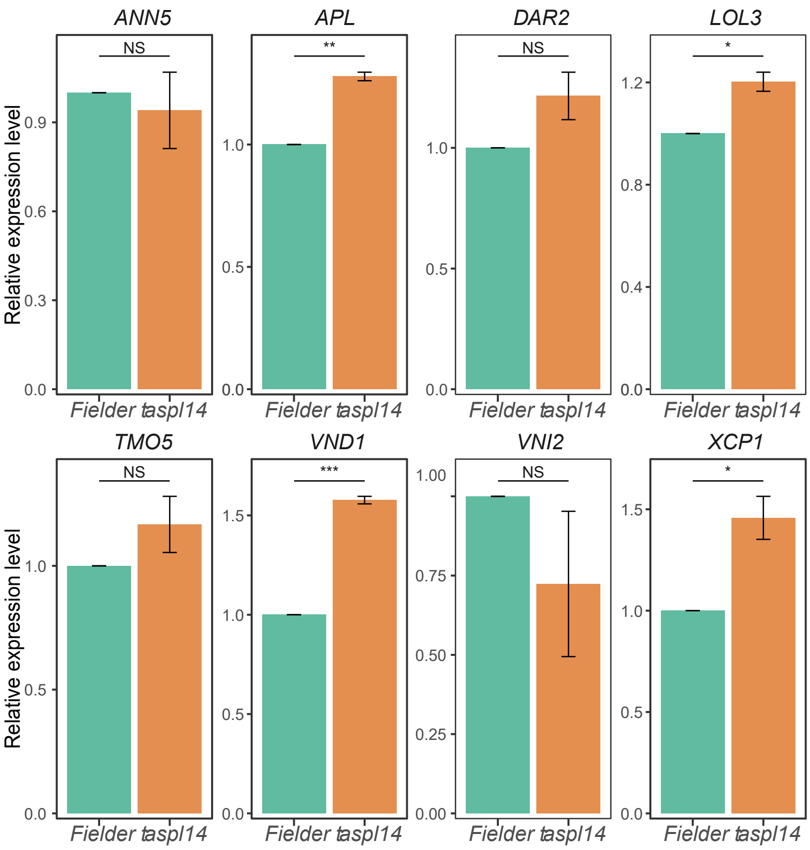

Fig. S13 Relative expression of marker genes of companion cells, protophloem and protoxylem in *taspl14* line 5. (* *p*-value < 0.05, ** *p*-value < 0.01, *** *p*-value < 0.001, ns indicate no significant difference).

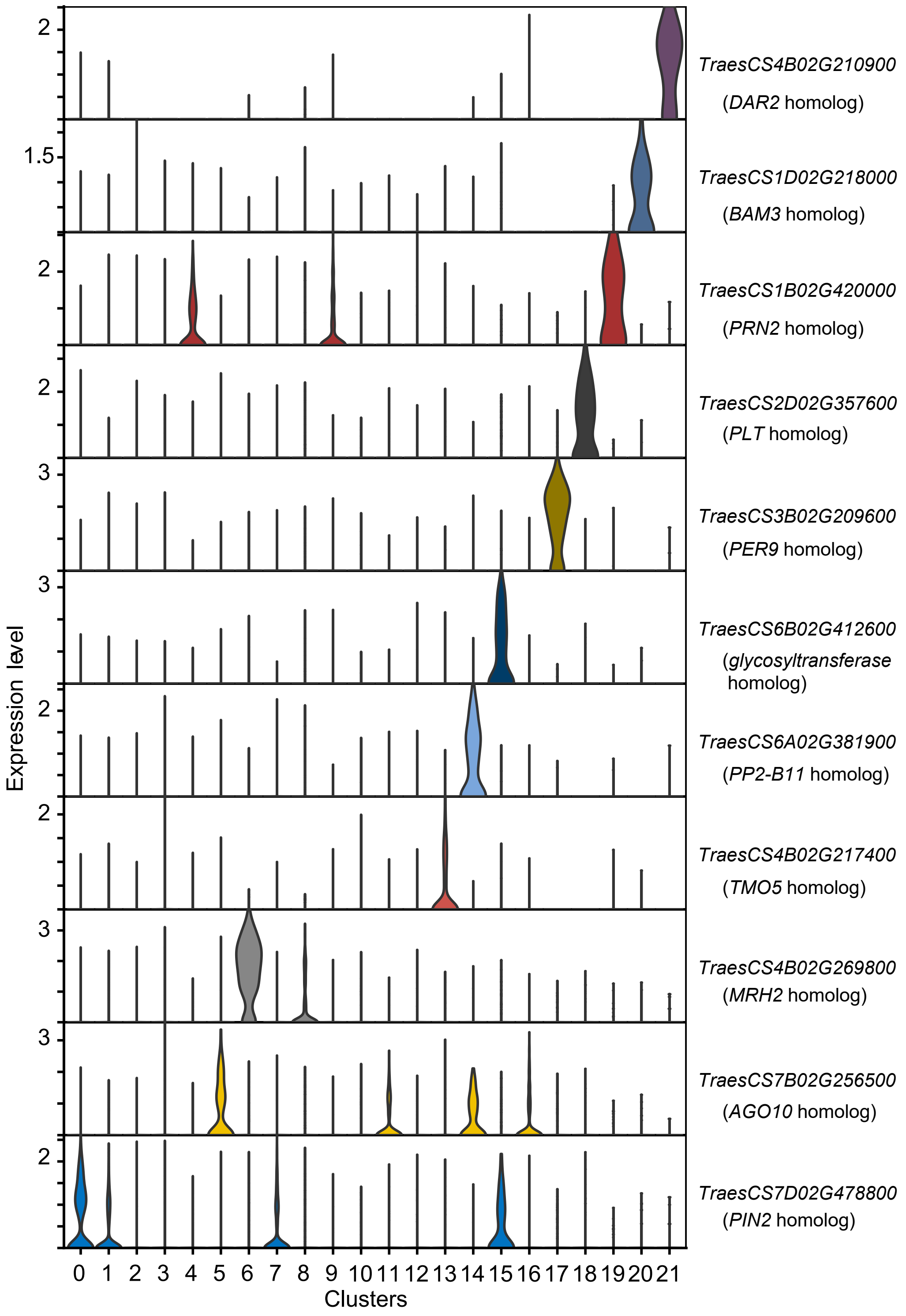

Fig. S14 Homologs of conservative genes between *Arabidopsis* and wheat root specifically expressed in corresponding wheat root cell clusters.
