# Supplementary material for: Asymmetric gene expression and cell-type-specific regulatory networks in the root of bread wheat revealed by single-cell multiomics analysis
Source: Genome Biol. 2023 Apr 4;24:65. doi: 10.1186/s13059-023-02908-x (PMC10074895; doi:10.1186/s13059-023-02908-x)
Supplement: Supplementary file 4 — Additional file 4. Supplementary Note. [file 13059_2023_2908_MOESM4_ESM.doc]

Supplementary Note

Homologs genes of *ROOT MERISTEM GROWTH FACTOR 1* (*RGF1*), *ROOT MERISTEM GROWTH FACTOR* 3 (*RGF3*) and *PLETHORA 1* (*PLT1*) expressed in cluster 18. All of these genes are key regulators for the maintenance of stem cell niche identity . Thus, we annotated cluster 18 as stem cell niche. Homologs for meristematic phase transition related genes and *Phospholipase D* (*PLD*) homologous genes for auxin signaling are enriched in cluster 2. *PLD* can control the root meristem size by regulating the PIN-FORMED 1 (PIN1) phosphorylation status and polar localization, as well as the subsequent auxin distribution at root meristem . Taking all the clues together, the cluster 2 is annotated as meristem. As cluster 7 is adjacent to cluster 2, the majority of the genes in cluster 7 are also enriched during the meristematic phase, the cluster 7 is assumed to be another meristem in the root tip. For reference, we have also tabulated our data in Table S2. We designate cluster 3 as the proximal meristem based on the appearance of homologs comprising meristem-related genes and the auxin transporter genes *PIN1* and *PIN4* for vascular pattern . An obvious common signature of the above cluster 2, 3 and 7 is that, the DNA replication and chromatin organization related genes were highly enriched in these cell types, which indicates their meristematic activity . Several homologs of *ARGONAUTE 10* (*AGO10*) that is vascular initial cell related and auxin related genes for vascular pattern co-express in cluster 5, indicate the provascular cell identity of cluster 5 . Cluster 13 is protoxylem with conservative homologs of *TARGET OF MONOPTEROS 5* (*TMO5*) , *VASCULAR RELATED NAC-DOMAIN PROTEIN 1* (*VND1*) and *XYLEM CYSTEINE PEPTIDASE 1* (*XCP1*) highly expressed . Homologs of lignin and xylan biosynthetic genes and homologs of xylem marker genes *IRREGULAR XYLEM 9* (*IRX9*) are marker genes with high and specific expression profile in cluster 11, all of which suggest this cluster is metaxylem . The homologs of protophloem specific *ALTERED PHLOEM DEVELOPMENT* (*APL*) show highest expression level in cluster 16. APL is a key regulator for phloem development . The homologs of *VND-INTERACTING 2* (*VNI2*) also express in cluster 16. VNI2 can change xylem vessel identity by interacting with VND7 . Therefore, we designate cluster 16 as protophloem cells. Similarly, cluster 21 is annotated as companion cells since the homologs of *DA1-RELATED PROTEIN2* (*DAR2*) exhibit highest expression levels in cluster 21. DAR2 is specific in companion cells and responsible for phloem unloading at the early stage to regulate phloem development in *Arabidopsis* . Transcription of *APL* as well as homologs of metabolite synthesis and transport related genes, such as *CALCIUM-DEPENDENT PROTEIN KINASE 29* (*CPK29*), *SUCROSE TRANSPORTER 3* (*SUC3*), *SULTR-like P Distribution Transporter* (*SPDT*), *SPERMIDINE SYNTHASE* (*SPMS*), *THIAMIN PYROPHOSPHOKINASE1* (*TPK1*) and *ONSET OF LEAF DEATH 5* (*OLD5*) are also enriched in cluster 21. It is reported that Annexin (ANN) proteins can regulate the post-phloem sugar unloading in sieve elements, a phloem specific cell type . The homologs of *TPS1* (*TREHALOSE-6-PHOSPHATE SYNTHASE 1*) were also identified as the marker genes of cluster 14. Recently, *TPS1* has been demonstrated to be essential for signaling of sucrose status and plant viability, which is expressed in phloem-loading zone and root vasculature . Moreover, phloem proteins 2 (PP2) could depose in sieve pore and homologs of *PP2* and vascular genes were expressed in cluster 14. All of these suggested an immature sieve element identity of cluster 14. Cluster 9 expresses many homologs for auxin and lipid metabolic related genes, including homologs of *long-chain fatty acid-CoA ligase* (*AT4G23850*) and *lipoxygenase* (*LOX*). The previous studies showed that auxin can induce the initiation of lateral root primordial in pericycle . Meanwhile, the *very-long-chain fatty acid* (*VLCFA*) precisely restricted cell proliferation at the initiating zone of lateral root primordial, induced by auxin . The *LOX* genes are also important for the formation of lateral root primordial . Interestingly, there are many homologs of casparian strip lignification related genes which were also highly expressed in cluster 9. It is reported that, both cells of pericycle and endodermis participate in the formation of the lateral root primordial in cereals .This is different from the situation in *Arabidopsis*. Moreover, the lateral root primordia in cereals always emerged at the pericycle beside phloem pole . Therefore, we defined the cluster 9 as the phloem pole pericycle (PPP). Recently, gene expression analysis suggested a specific function of pericycle cells for water transport, sulphur assimilation and jasmonic acid biosynthesis in rice . Our data shows that homologs of water transport proteins *PLASMA MEMBRANE INTRINSIC PROTEINs* (*PIPs*) and sulfate transporter are specifically expressed in cluster 4 . Based on this demonstration, we assign the cluster 4 as xylem pole pericycle cell type (XPP), which is relative to the phloem pole pericycle (PPP) . We also found that the cluster 4 and 9 originated from Cluster 1(Figure 1b). Homologs of cell elongation related genes present the high variable expression in cluster 1, including the BR and auxin related genes, *PLD* genes and cell wall synthesis related genes like the homologs of CELLULOSE SYNTHASE family proteins . The previous study shows that, before LRP initiation, the pericycle cells require five or six times division after differentiating from the pericycle initial cell . Meanwhile, the pericycle cell length has been demonstrated an important role in LRP initiation . The homologous gene of *VACUOLAR PHOSPHATE TRANSPORTER 1* (*VPT1*) is also high expression in cluster 1 and essential for phosphate homeostasis, which affects the lateral root development in plants . Therefore, we proposed the cluster 1 as an immature pericycle cells (IPC) undergoing cell elongation. we compared all the marker genes of cluster 0 and cluster 12 with the enriched marker genes of epidermis and cortex in other species. The venn diagram showed that the marker genes of cluster 12 are more overlapped with marker genes of cortex cells rather than epidermis in *Arabidopsis* and maize [18, 47]. Nevertheless, contradictory conclusion was obtained when compared with rice [48] (Supplementary Note Figure 1A). As for cluster 0, it shares epidermis marker with other species but there are also quite some cortex markers shared (Supplementary Note Figure 1 B). These results indicated both cluster 0 and cluster 12 possessed the characters of epidermis and cortex. There are homologs of *DUF642* showing high expression in cluster 0. Most DUF642 proteins locate at the cell wall and can regulate the pectin methyl esterase’s activity and cell wall properties [49]. DUF642 L-GALL RESPONSIVE GENE 2 (DGR2) was identified as cortex enriched gene in *Arabidopsis* and *Lotus japonicas* . However, other DUF642 proteins are also expressed in root epidermis in *Arabidopsis*, including BIIDXI (BDX), At5g11420, At2g41800 (TEB), and At3g08030 [49]. Auxin efflux carrier PIN2 also shows specific and high expression in cluster 0. PIN2 has been demonstrated opposite locations in epidermis and cortex [51]. We performed RNA *in situ* hybridization for marker genes of cluster 0, including homologs of *EXPANSIN 11* (*EXPA11*) *KANADI 1* (*KAN1*) *AUXIN-INDUCED IN ROOT CULTURES 3* (*AIR3*). The results show that *KAN1* is expressed in both epidermis and cortex, while *EXPA11* and *AIR3* mainly presented in epidermis with weaker expression in cortex (Supplementary Note Figure 2). Therefore, we annotate the cluster 0 as epidermis/cortex. Cluster 12 expresses homologs of flavonoid biosynthetic genes, auxin efflux transporters of *ATP-BINDING CASSETTE B* (*ABCB*), *CYTOKININ RESPONSE 1* (*CRE1*) and *MYB DOMAIN PROTEIN 86* (*MYB86*). It is reported that the flavonoids can accumulate in epidermis and cortex of *Arabidopsis* root, as well as dividing cells in other species . The CRE1 can induce the expression of flavonoid biosynthetic genes and can regulate the local auxin accumulation in inner cortex, which is necessary for the cortical cell division and nodulation in root inner cortex . Interestingly, the MYB86 is also associated with the flavonoid biosynthesis in other species, such as fruits of *Fragaria vesca*, rabbiteye, blueberry and tea plants (*Camellia sinensis* L.) . RNA *in situ* hybridization for marker genes of cluster 12 showed that WALL-ASSOCIATED KINASE 2 (WAK2) and MYB86 were expressed in cortex and epidermis, but RIBONUCLEASE 1 (RNS1) was strongly expressed in epidermis with signal in cortex been detected (Supplementary Note Figure 2). Therefore, we also annotate the cluster 12 as the epidermis/cortex in wheat root. As endodermis always comes along with a thickened casparian, the cluster 10 and 17 are endodermis cells with high expression of many endodermis related homologous genes [60]. A large number of genes regulating root hair differentiation and genes for auxin polar transportation were highly expressed in cluster 6 (Supplementary Note Figure 3). Auxin is also important for root hair development [61]. Based on the above facts, cluster 6 can be defined as root hair cells. There are several root hairs related genes enriched in cluster 8 (Table S2). However, no cluster specific genes were identified for cluster 8 (Figure 1d). Since cluster 8 is adjacent to cluster 0, 6 and 12 in the three-dimensional UMAP scatterplots (Supplementary data 1), we used the venn diagram to analyze the overlaps that cluster 8 with cluster 0, 6 and 12. The result indicated that cluster 8 is similar with both of cluster 6 and 12 (Supplementary Note Figure 4). Based on several root hairs related genes enriched in cluster 8 and the venn diagram result, we annotated the cluster 8 as epidermis/root hair. The cluster 15 expressed many homologs of late embryogenesis abundant protein (*LEA*) (*At3g19430*) gene family. These proteins are highly hydrophilic and thermo-stable and play key roles in the desiccation tolerance for terrestrial plants . It has been reported that LEA protein homolog zmRCPl and zmRCP2 were expressed in outermost cells of root cap in maize . Interestingly, GO annotation of marker genes shows that many saccharides metabolism related genes are expressed in cluster 15 (Supplementary Note Figure 5), such as *PHOSPHOMANNOSE ISOMERASE 1* (*PMI1*), *O-glucosyltransferase*, *FUCOSYLTRANSFERASE 1* (*FT1*), *SUGAR TRANSPORTER PROTEIN 7* (*STP7*), *REVERSIBLY GLYCOSYLATED POLYPEPTIDE 2* (*RGP2*), *REVERSIBLY GLYCOSYLATED POLYPEPTIDE 1* (*RGP1*), *GLUCOSE-6-PHOSPHATE/PHOSPHATE TRANSLOCATOR 2* (*GPT2*), *UDP-D-glucose 4-epimerase*, *GDP-D-MANNOSE 3* (*GME*), *ROOT HAIR DEFECTIVE 1* (*RHD1*), *CYTOKINESIS DEFECTIVE 1* (*CYT1*), and *SUGAR TRANSPORTER 1* (*STP1*) (Table S2). Many studies proved that root cap changed into secretory cells at their distal terminal (outer root cap or older root cap, which includes cells of distal root cap and lateral root cap), and produce mucilage contained saccharides and polysaccharide . BEARSKIN1 (BRN1) and BRN2 have been demonstrated to be necessary for the mucilage accumulation around root cap in *Arabidopsis* . Moreover, the expression of *BEARSKIN1* (*BRN1*) and *BRN2* is restricted to the outermost root cap layers (distal root cap and lateral root cap) and control the maturation of root cap . Here, we found that the *BRN2* is specifically expressed in cluster 15 (Supplementary Note Figure 6). Therefore, we infer the cluster 15 is root cap, including distal root cap and lateral root cap. The homologs of osmotic stress related gene *INTEGRIN-LINKED KINASE1* (*ILK1*) is also expressed in cluster 15 . We have noticed that later root cap plays a key role in root hydrotropism, which may be important for wheat root to adapt the semiarid soil . The aggregation of hydrophilic *LEA* and osmotic stress responsive *ILK1* in cluster 15 may support a specialized function for hydrotropism of cluster 15 which is important for wheat root to adapt to the arid soil habitat. It is interesting that cluster 20 cells expressed homologous genes of *β-AMYLASE* 3 (*BAM3*) for starch catabolism. The sedimentation of amyloplasts filled with starch can mediate gravity sensing in root columella . Thus, we propose that the cluster 20 is columella. Cluster 19 is featured by many defense related transcriptions, which may indicate the role of this cluster in wheat defense. The specialized immune related cells actually existed in root, namely root border cells. Root border cells separated from root cap will become uniquely differentiated cells with many defense proteins to fight against soil-borne pathogenic bacteria and fungus . Thereby, cluster 19 is a cell type similar to root border cells that dissociate immediately from root cap (Fig. S4).

**References**

1. Aida M, Beis D, Heidstra R, Willemsen V, Blilou I, Galinha C, et al. The *PLETHORA* genes mediate patterning of the *Arabidopsis* root stem cell niche. *Cell*. 2004;119:109–120.

2. Lu X, Shi H, Ou Y, Cui Y, Chang J, Peng L, et al. RGF1-RGI1, a peptide-receptor complex, regulates *Arabidopsis* root meristem development via a MAPK signaling cascade. *Mol Plant*. 2020,13:1594–1607.

3. Matsuzaki Y, Ogawa-Ohnishi M, Mori A, Matsubayashi Y. Secreted peptide signals required for maintenance of root stem cell niche in *Arabidopsis*. *Science*. 2010;329:1065–1067.

4. Gao HB, Chu YJ, Xue HW. Phosphatidic acid (PA) binds PP2AA1 to regulate PP2A activity and PIN1 polar localization. *Mol Plant*. 2013;6:1692–1702.

5. Liu Y, Xu M, Liang N, Zheng Y, Yu Q, Wu S. Symplastic communication spatially directs local auxin biosynthesis to maintain root stem cell niche in *Arabidopsis*. *Proc Natl Acad Sci U S A*. 2017;114:4005–4010.

6. Zhou W, Wei L, Xu J, Zhai Q, Jiang H, Chen R, et al. *Arabidopsis* tyrosylprotein sulfotransferase acts in the auxin/PLETHORA pathway in regulating postembryonic maintenance of the root stem cell niche. *Plant Cell*. 2010;22:3692–3709.

7. Blilou I, Xu J, Wildwater M, Willemsen V, Paponov I, Friml J, et al. The PIN auxin efflux facilitator network controls growth and patterning in *Arabidopsis* roots. *Nature*. 2005;433:39–44.

8. Ruzicka K, Simaskova M, Duclercq J, Petrasek J, Zazimalova E, Simon S, et al. Cytokinin regulates root meristem activity via modulation of the polar auxin transport. *Proc Natl Acad Sci U S A*. 2009;106:4284–4289.

9. Sozzani R, Iyer-Pascuzzi A. Postembryonic control of root meristem growth and development. *Curr Opin Plant Biol*. 2014;17:7–12.

10. Zhang T, Zhang W, Jiang J. Genome-wide nucleosome occupancy and positioning and their impact on gene expression and evolution in plants. *Plant Physiol*. 2015;168:1406–1416.

11. Hashimura Y, Ueguchi C. The *Arabidopsis* *MERISTEM DISORGANIZATION 1* gene is required for the maintenance of stem cells through the reduction of DNA damage. *Plant J*. 2011;68:657–669.

12. Apelt F, Mavrothalassiti E, Gupta S, Machin F, Olas JJ, Annunziata MG, et al. Shoot and root single cell sequencing reveals tissue- and daytime-specific transcriptome profiles. *Plant Physiol*. 2022;188:861–878.

13. Tucker MR, Hinze A, Tucker EJ, Takada S, Jurgens G, Laux T. Vascular signalling mediated by ZWILLE potentiates WUSCHEL function during shoot meristem stem cell development in the *Arabidopsis* embryo. *Development*. 2008;135:2839–2843.

14. Zhou Y, Honda M, Zhu H, Zhang Z, Guo X, Li T, et al. Spatiotemporal sequestration of *miR165/166* by *Arabidopsis* Argonaute10 promotes shoot apical meristem maintenance. *Cell Rep*. 2015;10:1819–1827.

15. Truernit E, Bauby H, Belcram K, Barthelemy J, Palauqui JC. OCTOPUS, a polarly localised membrane-associated protein, regulates phloem differentiation entry in *Arabidopsis thaliana*. *Development*. 2012;139:1306–1315.

16. Konishi M, Donner TJ, Scarpella E, Yanagisawa S. MONOPTEROS directly activates the auxin-inducible promoter of the Dof5.8 transcription factor gene in *Arabidopsis thaliana* leaf provascular cells. *J Exp Bot*. 2015;66:283–291.

17. Holding DR, Springer PS. The vascular prepattern enhancer trap marks early vascular development in *Arabidopsis*. *Genesis*. 2002;33:155–159.

18. Zhang TQ, Xu ZG, Shang GD, Wang JW. A single-cell RNA sequencing profiles the developmental landscape of *Arabidopsis* root. *Mol Plant*. 2019;12:648–660.

19. Tan TT, Endo H, Sano R, Kurata T, Yamaguchi M, Ohtani M, et al. Transcription factors VND1-VND3 contribute to cotyledon xylem vessel formation. *Plant Physiol*. 2018;176:773–789.

20. Avci U, Earl PH, Ismail IO, Beers EP, Haigler CH. Cysteine proteases XCP1 and XCP2 aid micro-autolysis within the intact central vacuole during xylogenesis in *Arabidopsis* roots. *Plant J*. 2008;56:303–315.

21. Bonke M, Thitamadee S, Mahonen AP, Hauser MT, Helariutta Y. APL regulates vascular tissue identity in *Arabidopsis*. *Nature*. 2003;426:181–186.

22. Turco GM, Rodriguez-Medina J, Siebert S, Han D, Valderrama-Gomez MA, Vahldick H, et al. Molecular mechanisms driving switch behavior in xylem cell differentiation. *Cell Rep*. 2019;28:342–351.

23. Peng Y, Ma W, Chen L, Yang L, Li S, Zhao H, et al: Control of root meristem size by DA1-RELATED PROTEIN2 in *Arabidopsis*. *Plant Physiol*. 2013;161:1542–1556.

24. Wang J, Song J, Clark G, Roux SJ. ANN1 and ANN2 function in post-phloem sugar transport in root tips to affect primary root growth. *Plant Physiol*. 2018;178:390–401.

25. Fichtner F, Olas JJ, Feil R, Watanabe M, Krause U, Hoefgen R, et al. Functional features of TREHALOSE-6-PHOSPHATE SYNTHASE1, an essential enzyme in *Arabidopsis*. *Plant Cell*. 2020;32:1949–1972.

26. Vincill ED, Clarin AE, Molenda JN, Spalding EP. Interacting glutamate receptor-like proteins in phloem regulate lateral root initiation in *Arabidopsis*. *Plant Cell*. 2013;25:1304–1313.

27. Dubrovsky JG, Sauer M, Napsucialy-Mendivil S, Ivanchenko MG, Friml J, Shishkova S, et al. Auxin acts as a local morphogenetic trigger to specify lateral root founder cells. *Proc Natl Acad Sci U S A*. 2008;105:8790–8794.

28. Lv B, Wei K, Hu K, Tian T, Zhang F, Yu Z, et al. MPK14-mediated auxin signaling controls lateral root development via ERF13-regulated very-long-chain fatty acid biosynthesis. *Mol Plant*. 2021;14:285–297.

29. Boutte Y, Jaillais Y. Metabolic Cellular Communications: Feedback mechanisms between membrane lipid homeostasis and plant development. *Dev Cell*. 2020;54:171–182.

30. Trinh DC, Lavenus J, Goh T, Boutte Y, Drogue Q, Vaissayre V, et al. PUCHI regulates very long chain fatty acid biosynthesis during lateral root and callus formation. *Proc Natl Acad Sci U S A*. 2019;116:14325–14330.

31. Vellosillo T, Martinez M, Lopez MA, Vicente J, Cascon T, Dolan L, et al. Oxylipins produced by the 9-lipoxygenase pathway in *Arabidopsis* regulate lateral root development and defense responses through a specific signaling cascade. *Plant Cell*. 2007;19:831–846.

32. Yu P, Gutjahr C, Li C, Hochholdinger F. Genetic control of lateral root formation in cereals. *Trends Plant Sci*. 2016;21:951–961.

33. Aubry S, Smith-Unna RD, Boursnell CM, Kopriva S, Hibberd JM. Transcript residency on ribosomes reveals a key role for the *Arabidopsis thaliana* bundle sheath in sulfur and glucosinolate metabolism. *Plant J*. 2014;78:659–673.

34. Hua L, Stevenson SR, Reyna-Llorens I, Xiong H, Kopriva S, Hibberd JM. The bundle sheath of rice is conditioned to play an active role in water transport as well as sulfur assimilation and jasmonic acid synthesis. *Plant J*. 2021;107:268–286.

35. Kirschner S, Woodfield H, Prusko K, Koczor M, Gowik U, Hibberd JM, et al. Expression of SULTR2;2, encoding a low-affinity sulphur transporter, in the *Arabidopsis* bundle sheath and vein cells is mediated by a positive regulator. *J Exp Bot*. 2018;69:4897–4906.

36. Shahan R, Hsu CW, Nolan TM, Cole BJ, Taylor IW, Greenstreet L, et al: A single-cell *Arabidopsis* root atlas reveals developmental trajectories in wild-type and cell identity mutants. *Dev Cell*. 2022;57(4):543–560.

37. Vukasinovic N, Wang Y, Vanhoutte I, Fendrych M, Guo B, Kvasnica M, et al. Local brassinosteroid biosynthesis enables optimal root growth. *Nat Plants*. 2021;7:619–632.

38. Lin DL, Yao HY, Jia LH, Tan JF, Xu ZH, Zheng WM, et al. Phospholipase D-derived phosphatidic acid promotes root hair development under phosphorus deficiency by suppressing vacuolar degradation of PIN-FORMED2. *New Phytol*. 2020;226:142–155.

39. Potocky M, Elias M, Profotova B, Novotna Z, Valentova O, Zarsky V. Phosphatidic acid produced by phospholipase D is required for tobacco pollen tube growth. *Planta*. 2003;217:122–130.

40. Du M, Spalding EP, Gray WM. Rapid auxin-mediated cell expansion. *Annu Rev Plant Biol*. 2020;71:379–402.

41. Li L, Verstraeten I, Roosjen M, Takahashi K, Rodriguez L, Merrin J, et al. Cell surface and intracellular auxin signalling for H^+^ fluxes in root growth. *Nature*. 2021;599:273–277.

42. Dubrovsky JG, Doerner PW, Colon-Carmona A, Rost TL. Pericycle cell proliferation and lateral root initiation in *Arabidopsis*. *Plant Physiol*. 2000;124:1648–1657.

43. Alarcon MV, Salguero J, Lloret PG. Auxin modulated initiation of lateral roots is linked to pericycle cell length in maize. *Front Plant Sci*. 2019;10:11.

44. Perez-Torres CA, Lopez-Bucio J, Cruz-Ramirez A, Ibarra-Laclette E, Dharmasiri S, Estelle M, et al. Phosphate availability alters lateral root development in *Arabidopsis* by modulating auxin sensitivity via a mechanism involving the TIR1 auxin receptor. *Plant Cell*. 2008;20:3258–3272.

45. Luan M, Liu J, Liu Y, Han X, Sun G, Lan W, et al. Vacuolar phosphate transporter 1 (VPT1) affects arsenate tolerance by regulating phosphate homeostasis in *Arabidopsis*. *Plant Cell Physiol*. 2018;59:1345–1352.

46. Liu J, Yang L, Luan M, Wang Y, Zhang C, Zhang B, et al. A vacuolar phosphate transporter essential for phosphate homeostasis in *Arabidopsis*. *Proc Natl Acad Sci U S A*. 2015;112:E6571–6578.

47. Ortiz-Ramirez C, Guillotin B, Xu X, Rahni R, Zhang S, Yan Z, et al: Ground tissue circuitry regulates organ complexity in maize and *Setaria*. *Science*. 2021;374:1247–1252.

48. Zhang TQ, Chen Y, Liu Y, Lin WH, Wang JW. Single-cell transcriptome atlas and chromatin accessibility landscape reveal differentiation trajectories in the rice root. *Nat Commun*. 2021;12:2053.

49. Cruz-Valderrama JE, Gomez-Maqueo X, Salazar-Iribe A, Zuniga-Sanchez E, Hernandez-Barrera A, Quezada-Rodriguez E, et al. Overview of the role of cell wall DUF642 proteins in plant development. *Int J Mol Sci*. 2019;20(13):3333.

50. Sun Z, Jiang S, Wang D, Li L, Liu B, Ran Q, et al. Single-cell RNA-seq of *Lotus japonicus* provide insights into identification and function of root cell types of legume. *J Integr Plant Biol*. 2022. https://doi.org/10.1111/jipb.13435.

51. Rigo G, Ayaydin F, Tietz O, Zsigmond L, Kovacs H, Pay A, et al. Inactivation of plasma membrane-localized CDPK-RELATED KINASE5 decelerates PIN2 exocytosis and root gravitropic response in *Arabidopsis*. *Plant Cell*. 2013;25:1592–1608.

52. Wasson AP, Ramsay K, Jones MGK, Mathesius U. Differing requirements for flavonoids during the formation of lateral roots, nodules and root knot nematode galls in *Medicago truncatula*. *New Phytol*. 2009;183:167–179.

53. Djordjevic MA, Mathesius U, Arioli T, Weinman JJ, Gartner E. Chalcone synthase gene expression in transgenic subterranean clover correlates with localised accumulation of flavonoids. *Aust J Plant Physiol*. 1997;24:119–132.

54. Morris AC, Djordjevic MA. The *Rhizobium leguminosarum biovar trifolii* ANU794 induces novel developmental responses on the subterranean clover cultivar *Woogenellup*. *Mol Plant Microbe Interact*. 2006;19:471–479.

55. Saslowsky D, Winkel-Shirley B. Localization of flavonoid enzymes in *Arabidopsis* roots. *Plant J*. 2001;27:37–48.

56. Gamas P, Brault M, Jardinaud MF, Frugier F. Cytokinins in symbiotic nodulation: when, where, what for? *Trends Plant Sci*. 2017;22:792–802.

57. Zhang YC, Li WJ, Dou YJ, Zhang JX, Jiang GH, Miao LX, et al. Transcript quantification by RNA-Seq reveals differentially expressed genes in the red and yellow fruits of *Fragaria vesca*. *Plos One*. 2015;10(12):e0144356.

58. Gao X, Wang L, Zhang H, Zhu B, Lv G, Xiao J. Transcriptome analysis and identification of genes associated with floral transition and fruit development in rabbiteye blueberry (*Vaccinium ashei*). *PLoS One*. 2021;16:e0259119.

59. Ye JH, Lv YQ, Liu SR, Jin J, Wang YF, Wei CL, et al. Effects of light intensity and spectral composition on the transcriptome profiles of leaves in shade grown tea plants (*Camellia sinensis* L.) and regulatory network of flavonoid biosynthesis. *Molecules*. 2021;26(19):5836.

60. Geldner N. The endodermis. *Annu Rev Plant Biol* 2013;64:531–558.

61. Shibata M, Sugimoto K. A gene regulatory network for root hair development. *J Plant Res*. 2019;132:301–309.

62. Amara I, Capellades M, Ludevid MD, Pages M, Goday A. Enhanced water stress tolerance of transgenic maize plants over-expressing *LEA Rab28* gene. *J Plant Physiol*. 2013;170:864–873.

63. Wang Y, Xu H, Zhu H, Tao Y, Zhang G, Zhang L, et al. Classification and expression diversification of wheat dehydrin genes. *Plant Sci*. 2014;214:113–120.

64. Koubaa S, Brini F. Functional analysis of a wheat group 3 late embryogenesis abundant protein (TdLEA3) in *Arabidopsis thaliana* under abiotic and biotic stresses. *Plant Physiol Biochem*. 2020;156:396–406.

65. Magwanga RO, Lu P, Kirungu JN, Dong Q, Hu Y, Zhou Z, et al. Cotton *Late Embryogenesis Abundant* (*LEA2*) genes promote root growth and confer drought stress tolerance in transgenic *Arabidopsis thaliana*. *G3 (Bethesda)*. 2018;8:2781–2803.

66. Cheng Z, Zhang X, Yao W, Zhao K, Liu L, Fan G, et al. Genome-wide search and structural and functional analyses for *late embryogenesis-abundant* (*LEA*) gene family in poplar. *BMC Plant Biol*. 2021;21:110.

67. Matsuyama T, Yasumura N, Funakoshi M, Yamada Y, Hashimoto T. Maize genes specifically expressed in the outermost cells of root cap. *Plant Cell Physiol*. 1999;40:469–476.

68. Paull RE, Johnson CM, Jones RL. Studies on the secretion of maize root cap slime: I. Some properties of the secreted polymer. *Plant Physiol*. 1975;56:300–306.

69. Chaboud A, Rougier M. Identification and localization of sugar components of rice (*Oryza sativa* L.) root cap mucilage. *J Plant Physiol*. 1984;116:323–330.

70. Chaboud A. Isolation, purification and chemical-composition of maize root cap slime. *Plant Soil*. 1983;73:395–402.

71. Knee EM, Gong FC, Gao M, Teplitski M, Jones AR, Foxworthy A, et al. Root mucilage from pea and its utilization by rhizosphere bacteria as a sole carbon source. *Mol Plant Microbe Interact*. 2001;14:775–784.

72. Maeda K, Kunieda T, Tamura K, Hatano K, Hara-Nishimura I, Shimada T. Identification of periplasmic root-cap mucilage in developing columella cells of *Arabidopsis thaliana*. *Plant Cell Physiol*. 2019;60:1296–1303.

73. Kamiya M, Higashio SY, Isomoto A, Kim JM, Seki M, Miyashima S, et al. Control of root cap maturation and cell detachment by BEARSKIN transcription factors in *Arabidopsis*. *Development*. 2016;143:4063–4072.

74. Bennett T, van den TA, Sanchez-Perez GF, Campilho A, Willemsen V, Snel B, et al. SOMBRERO, BEARSKIN1, and BEARSKIN2 regulate root cap maturation in *Arabidopsis*. *Plant Cell*. 2010;22:640–654.

75. Brauer EK, Ahsan N, Dale R, Kato N, Coluccio AE, Pineros MA, et al. The Raf-like kinase ILK1 and the high affinity K+ transporter HAK5 are required for innate immunity and abiotic stress response. *Plant Physiol*. 2016;171:1470–1484.

76. Stinemetz C, Takahashi H, Suge H. Characterization of hydrotropism: the timing of perception and signal movement from the root cap in the agravitropic pea mutant ageotropum. *Plant Cell Physiol*. 1996;37:800–805.

77. Cassab GI, Eapen D, Campos ME. Root hydrotropism: an update. *Am J Bot*. 2013;100:14–24.

78. Yamazaki T, Miyazawa Y, Kobayashi A, Moriwaki T, Fujii N, Takahashi H. MIZ1, an essential protein for root hydrotropism, is associated with the cytoplasmic face of the endoplasmic reticulum membrane in *Arabidopsis* root cells. *Febs Letters*. 2012;586:398–402.

79. Takahashi H, Scott TK. Intensity of hydrostimulation for the induction of root hydrotropism and its sensing by the root cap. *Plant Cell Environ*. 1993;16:99–103.

80. Nakamura M, Nishimura T, Morita MT. Gravity sensing and signal conversion in plant gravitropism. *J Exp Bot*. 2019;70:3495–3506.

81. Su SH, Gibbs NM, Jancewicz AL, Masson PH. Molecular mechanisms of root gravitropism. *Curr Biol*. 2017;27:R964–R972.

82. Hawes M, Allen C, Turgeon BG, Curlango-Rivera G, Minh Tran T, Huskey DA, et al. Root border cells and their role in plant defense. *Annu Rev Phytopathol*. 2016;54:143–161.

83. Plancot B, Santaella C, Jaber R, Kiefer-Meyer MC, Follet-Gueye ML, Leprince J, et al. Deciphering the responses of root border-like cells of *Arabidopsis* and flax to pathogen-derived elicitors. *Plant Physiol*. 2013;163:1584–1597.


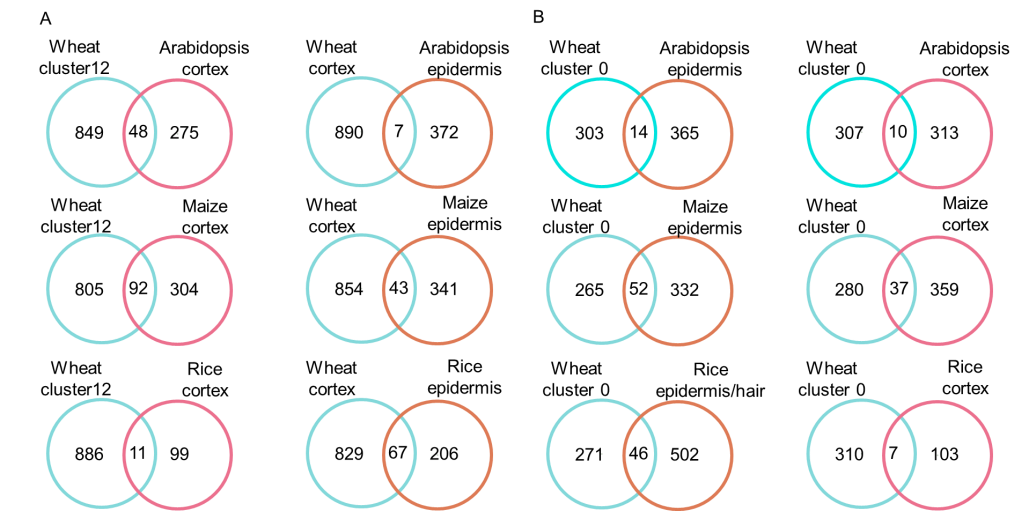


Supplementary Note Figure 1. Venn diagram showing the overlap between marker genes of cluster 12 and 0 with the enriched marker genes of epidermis and cortex in other species. (A) Overlap between marker genes of cluster 12 with the enriched marker genes of epidermis and cortex in other species. (B) Overlap between marker genes of cluster 0 with the enriched marker genes of epidermis and cortex in other species.


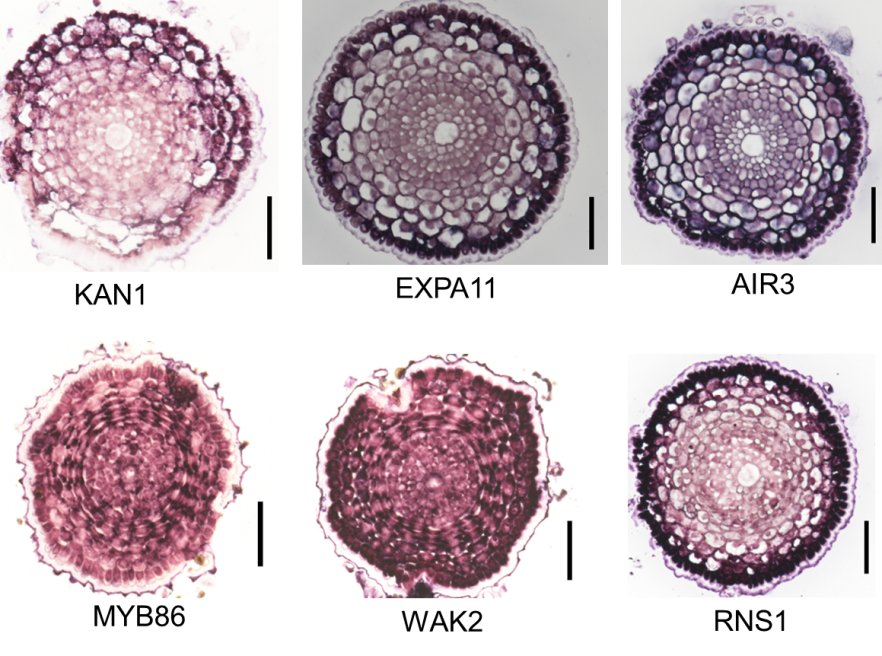


Supplementary Note Figure 2. RNA *in situ* hybridization shows the expression of marker genes of cluster 0 and 12 in root.


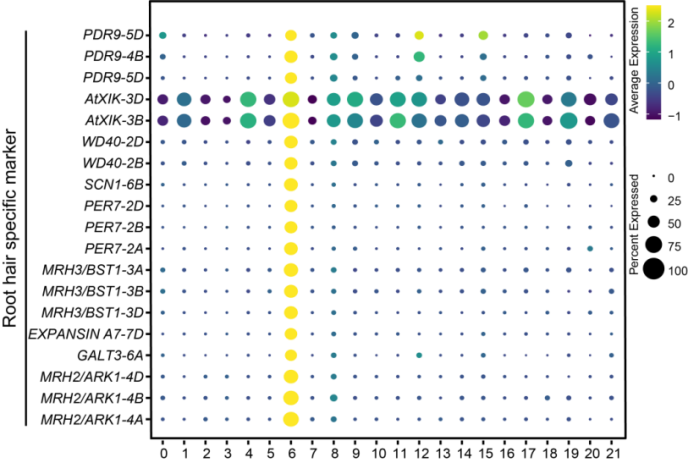


Supplementary Note Figure 3. The enriched root hair related genes in different clusters.


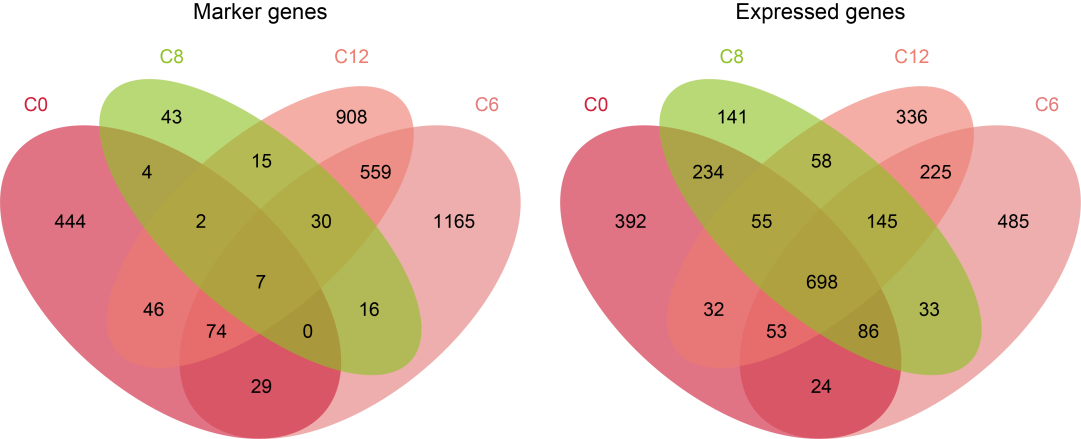


Supplementary Note Figure 4. Venn diagrams showing the overlapped genes between cluster 8 and cluster 6 and 12. C0, C6, C8 and C12 represented the cluster 0, 6, 8 and 12, respectively.


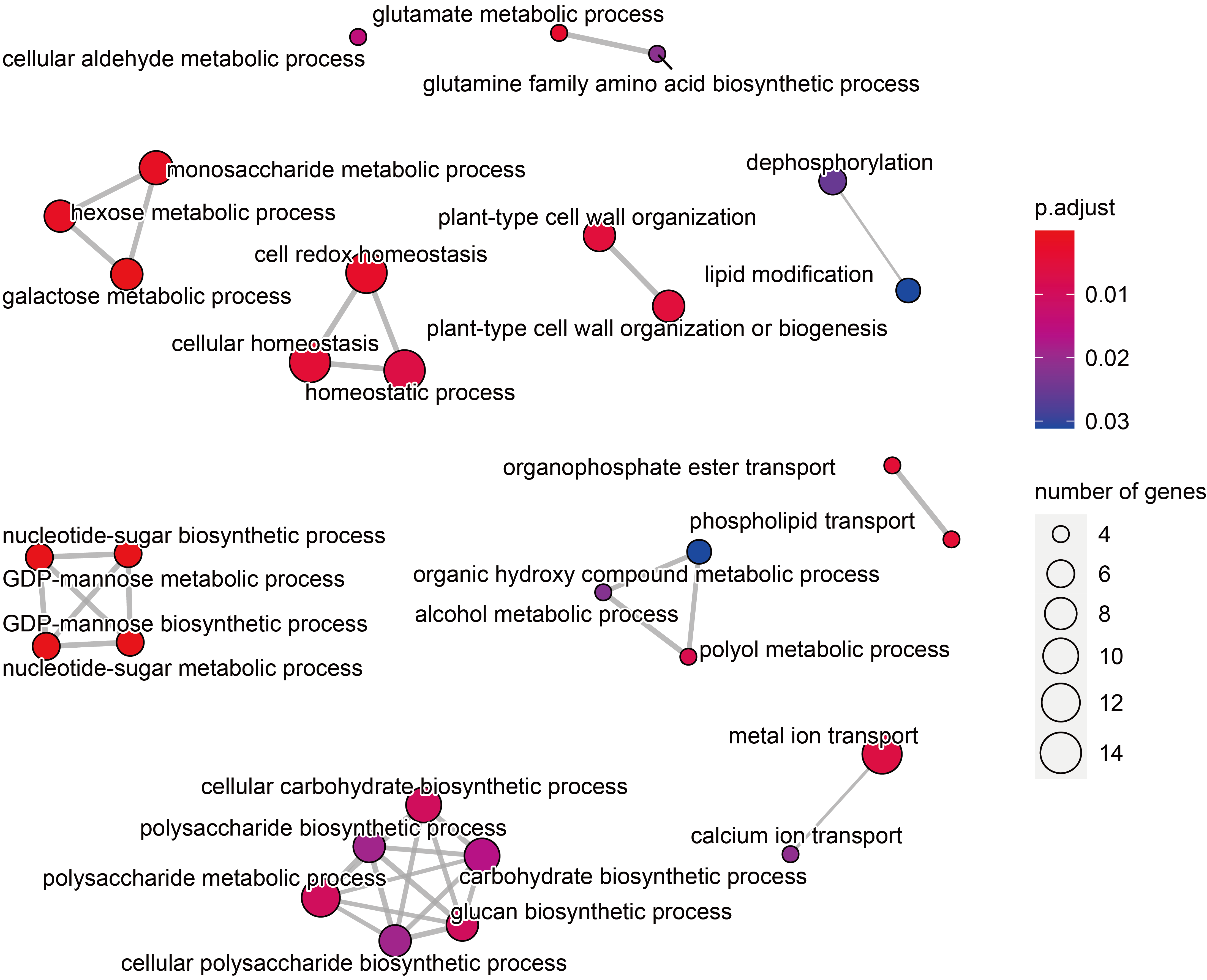


Supplementary Note Figure 5. GO annotation of marker genes of cluster 15.


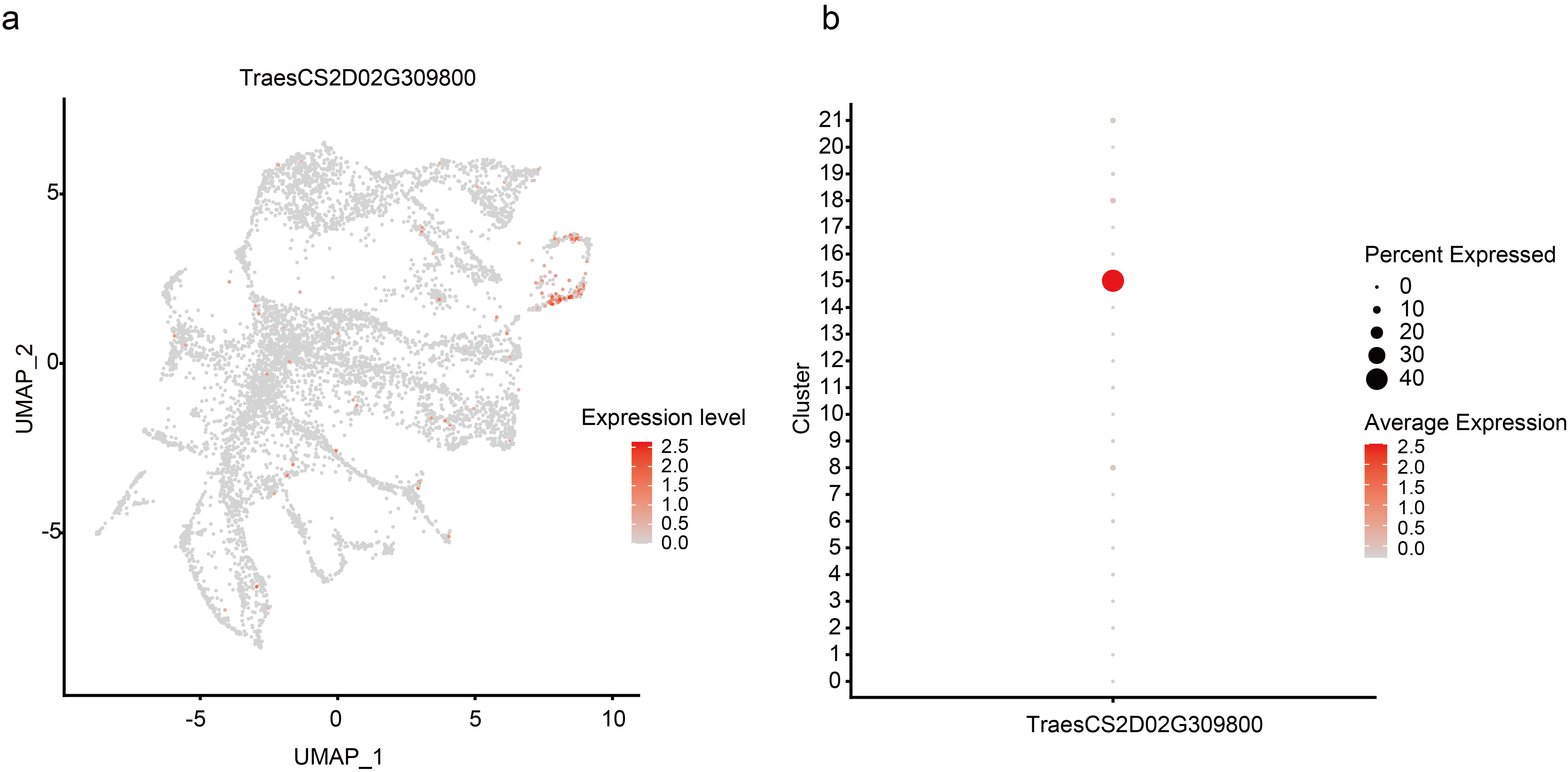


Supplementary Note Figure 6. The *BRN2* (*TraesCS2D02G309800*) is specifically expressed in cluster 15.
