# Supplementary material for: Asymmetric gene expression and cell-type-specific regulatory networks in the root of bread wheat revealed by single-cell multiomics analysis
Source: Genome Biol. 2023 Apr 4;24:65. doi: 10.1186/s13059-023-02908-x (PMC10074895; doi:10.1186/s13059-023-02908-x)
Supplement: Supplementary file 7 — Additional file 7. Review history. [file 13059_2023_2908_MOESM7_ESM.docx]

Review History

**First round of review**

**Reviewer 1**

**Were you able to assess all statistics in the manuscript, including the appropriateness of statistical tests used?**

**Yes, and I have assessed the statistics in my report.**

**Comments to author:**

Comments to the Author

As far as I know, this is the first paper describing a cell atlas of wheat roots at the single-cell resolution. The application of scRNA-seq and scATAC-seq technologies to study the differentiation trajectory of roots in this major crop is in time and novel. In general, the bioinformatic analysis part including cell clustering, annotation, and TRN meets the standard of the journal. The paper is well written and easy to follow. I have a few major points need to be clarified before I can recommend it for publication.

1. Pseudotime inference:

- Line 174 (Fig. 3a), authors used cluster 7 (meristem II), cluster 0 (epidermis), and cluster 12 (cortex) to infer the developmental trajectory of epidermis and cortex. However, based on the topology in Fig. 1B, cluster 7 is away from clusters 0 and 12. How could authors infer a continuum of cell differentiation toward epidermis and cortex?

-Line 176, "a subset of epidermal cells differentiated into cortex (cluster 12)". Do you mean cortex is derived from epidermal cells? I think this conclusion is totally wrong. In addition, cluster 12 does not link to any meristematic cell clusters on the UMAP. My guess is that the annotation of cluster 12 as cortex is not accurate. All the clusters (clusters 0, 6, 8 and 12) on the top belong to epidermal cells.

-Line 179, authors used clusters 2, 3, 7, 0, 12, 10, and 17 to infer the developmental trajectories for cortex and endodermis. Why cluster 0 (epidermis) was included? Again, cluster 12 is away from clusters 2, 3, 7, and 12. I think the correct order of the clusters used for the pseudo-time analysis is 2/7-3-10-17.

2. Mutant phenotype:

Line 284, Authors claimed that TaSPL14 is a key regulator in several vasculature cell types, including protoxylem (cluster 13), protophloem (cluster 16) and companion cells (cluster 21). Therefore, authors should examine whether all these cell types are altered in the taspl14 mutant. It should be noted that, based on the figure shown in fig. 5e, only the cell size in the vascular tissue is reduced. The root patterning is not affect.

Minor

- Line 114, "which was comparable with published single cell datasets from Arabidopsis". Which datasets? I could not references.

- Line 392, what is the D and DW buffer?

- Fig. 1c, please use the same code as (b) in the panel.

**Reviewer 2**

**Were you able to assess all statistics in the manuscript, including the appropriateness of statistical tests used?**

**Yes, and I have assessed the statistics in my report.**

**Comments to author:**

This is an interesting study that carries out single cell sequencing for wheat root using both snRNA-seq and snATAC-seq. Especially, the author investigated the asymmetrical transcription for wheat root development. The project provides import key regulators in root development of polyploid wheat.

Bread wheat included the AA, BB and DD genome. Asymmetric expression of homoeologs is important part for this study. However, the short reads were used for snRNA-seq and snATAC-seq in this study. Thus, there are many reads which could be mapped to both AA, BB and DD genome. How did the author calculate the expression of homoeologs? The author should provide detail description or custom pipeline in method section for phasing of reads for snRNA-seq and snATAC-seq to AA, BB and DD genome, respectively.

snRNA-seq can generate higher expression of long noncoding RNAs in snRNA-seq libraries than that of scRNA-seq. The author should provide the expression of long noncoding RNAs in 22 clusters since it is one of the advantage of snRNA-seq method. It will be interesting to find some cluster-specific marker long noncoding RNAs.

Line 135 The author can compare the 22 cluster-enriched genes from snRNA-seq in this study with that from published scRNA-seq/snRNA-seq root data in other species to present the percentage of overlapped orthologous genes for each cluster.

Line 136-138 The authors should provide expression information (heatmap) of more top enriched genes rather than only several representative cluster-specific marker genes (Fig. 1d) of 22 clusters.

Line 252 It's great interesting to see that snATAC-seq correlated with snRNA-seq. However, the result about snATAC-seq part is weak. The authors only provided general profile information for the association. Did the important cluster-specific marker genes present the association? Thus, more case study information should be provided for each cluster-specific marker genes to deeply mine snATAC-seq information.

The author should provide an online web server to facilitate the usage of the snRNA-seq and snATAC-seq datasets generated in this manuscript.

**Authors Response**

**Point-by-point responses to the reviewers’ comments:**

Reviewer #1: As far as I know, this is the first paper describing a cell atlas of wheat roots at the single-cell resolution. The application of scRNA-seq and scATAC-seq technologies to study the differentiation trajectory of roots in this major crop is in time and novel. In general, the bioinformatic analysis part including cell clustering, annotation, and TRN meets the standard of the journal. The paper is well written and easy to follow. I have a few major points need to be clarified before I can recommend it for publication.

*Response: Thank you very much for the positive comments. Yes, this is the first report of cell atlas in wheat. With this cell atlas, we hope to provide novel insights into root development and asymmetric gene transcription in wheat. We greatly appreciate the comments, questions and suggestion listed below. We found that they are very helpful and the whole work has been significantly improved by addressing these concerns.*

1. Pseudotime inference:

- Line 174 (Fig. 3a), authors used cluster 7 (meristem II), cluster 0 (epidermis), and cluster 12 (cortex) to infer the developmental trajectory of epidermis and cortex. However, based on the topology in Fig. 1B, cluster 7 is away from clusters 0 and 12. How could authors infer a continuum of cell differentiation toward epidermis and cortex?

*Response: Thank you for the question. We actually constructed a three-dimensional UMAP (3D-UMAP) scatterplots before we performed the trajectory analysis. From the 3D-UMAP, cluster 7 is close to cluster 0 and cluster 12 (Figure R1 and Supplementary data 1). We are sorry for the missed information in the previous manuscript and we have now included this information in the revised manuscript. We have added the sentence "To determine the spatial relationship among cell clusters, we also constructed the three-dimensional UMAP scatterplots for cell clusters (Supplementary data 1)." to line 137–138 in the manuscript. Please see line 137–138 and supplementary data 1. Regarding the differentiation of epidermis and cortex, we have omitted this part in the revised manuscript since we now have annotated the cluster 0 and cluster 12 as epidermis/cortex.*

Figure R1. The continuum of cluster 7, 0 and 12 in three-dimensional UMAP scatterplots.

-Line 176, "a subset of epidermal cells differentiated into cortex (cluster 12)". Do you mean cortex is derived from epidermal cells? I think this conclusion is totally wrong. In addition, cluster 12 does not link to any meristematic cell clusters on the UMAP. My guess is that the annotation of cluster 12 as cortex is not accurate. All the clusters (clusters 0, 6, 8 and 12) on the top belong to epidermal cells.

*Response: Thank you very much for the comments, which indeed helped us to more precisely interpret our data. The demonstration of "a subset of epidermal cells differentiated into cortex (cluster 12)" was based on the previous annotation and trajectory analysis. This sentence has been deleted in the revised manuscript due to the changed annotation of the cell clusters. Regarding the annotation of cluster 12 and other clusters, we defined the cell type by the function of the marker genes in the previous manuscript. However, inspired by the suggestions from the reviewer, we did more analysis and validation to confirm the cell types of the clusters. Firstly, we compared all the marker genes of cluster 0 and cluster 12 with the enriched marker genes of epidermis and cortex in other species. The venn diagram showed that the marker genes of cluster 12 are more overlapped with marker genes of cortex cells rather than epidermis in Arabidopsis and maize [**1**,* *2**]. Nevertheless, contradictory conclusion was obtained when compared with rice [**3**] (Supplementary Note Figure 1A). As for cluster 0, it shares epidermis marker with other species but there are also quite some cortex markers shared (Supplementary Note Figure 1B). These results indicated both cluster 0 and cluster 12 possessed the characters of epidermis and cortex. We further performed the RNA in situ hybridization to confirm the identity of the cell clusters. Homologs of KAN1, EXPA11 and AIR3 are the marker genes of cluster 0. KAN1 is expressed in both epidermis and cortex while EXPA11 and AIR3 are mainly expressed in epidermis with weaker expression in cortex. WAK2, MYB86, and RNS1 are the marker genes of cluster 12. WAK2 and MYB86 show clear expression in cortex and epidermis. RNS1 is strongly expressed in epidermis and signal in cortex can be also detected (Supplementary Note Figure 2). Based on all the above results and also the suggestions from the reviewers, to be more precise, we have now annotated both cluster 0 and 12 as epidermis/cortex. Since there are many root hairs related genes specifically enriched in cluster 6 (Supplementary Note Figure 3), thus we annotated cluster 6 as root hair. Several root hairs related genes are also presented in cluster 8 (Table S2), that was why we defined cluster 8 as root hair in the previous version of the manuscript although there are no clearly enriched marker genes in this cluster (Fig. 1d). However, when we re-analyzed our data following the comments from the reviewer, we found that cluster 8 shared more genes with cluster 6 and 12 (Supplementary Note Figure 4). Based on these results and the suggestions from the reviewers, we have now annotated the cluster 8 as epidermis/root hair. The above information has been included in the supplementary note.*

Supplementary Note Figure 1. Venn diagram showing the overlap between marker genes of cluster 12 and 0 with the enriched marker genes of epidermis and cortex in other species. (A) Overlap between marker genes of cluster 12 with the enriched marker genes of epidermis and cortex in other species. (B) Overlap between marker genes of cluster 0 with the enriched marker genes of epidermis and cortex in other species.

Supplementary Note Figure 2. RNA *in situ* hybridization showing the expression of marker genes of cluster 0 and 12 in root.

Supplementary Note Figure 3. The enriched root hair related genes in different clusters.

Supplementary Note Figure 4. Venn diagrams showing the overlapped genes between cluster 8 and cluster 6 and 12. C0, C6, C8 and C12 represented the cluster 0, 6, 8 and 12, respectively.

-Line 179, authors used clusters 2, 3, 7, 0, 12, 10, and 17 to infer the developmental trajectories for cortex and endodermis. Why cluster 0 (epidermis) was included? Again, cluster 12 is away from clusters 2, 3, 7, and 12. I think the correct order of the clusters used for the pseudo-time analysis is 2/7-3-10-17.

*Response: Thank you for the comments and suggestions. Since cluster 0 bridges cluster 12 and cluster 7 in the 3D-UMAP (sorry again for the missed 3D-UMAP in the last version of the manuscript) and we annotated cluster 12 as cortex, we included cluster 0 in the previous analysis. Now, we have annotated both clusters 0 and 12 as epidermis/cortex in the revised manuscript. The reviewer is right that the order for endodermis is 2/7-3-10-17 (Figure R2). In the revised manuscript, we used the clusters 2, 3, 7 (meristem), cluster 0 and 12 (epidermis/cortex), and cluster 10 and 17 (endodermis) to infer the developmental trajectories for epidermis/cortex and endodermis (Fig 3b).*

Figure R2. Differentiation trajectories of endodermis with cluster 2-7-3-10-17.

Fig. 3(b) Differentiated trajectory of epidermis/cortex and endodermis. Colors of dots are corresponding to cell clusters. The corresponding pseudo-time trajectory was shown on the right.

2. Mutant phenotype:

Line 284, Authors claimed that TaSPL14 is a key regulator in several vasculature cell types, including protoxylem (cluster 13), protophloem (cluster 16) and companion cells (cluster 21). Therefore, authors should examine whether all these cell types are altered in the taspl14 mutant. It should be noted that, based on the figure shown in fig. 5e, only the cell size in the vascular tissue is reduced. The root patterning is not affect.

*Response: Thank you for the nice suggestions. Following the suggestions, we prepared more slides with prolonged dewaxing time for the clearer details of the root cells between taspl14 mutant and wild type. In addition to the area of the vascular tissue, the number of protoxylem pores (cluster 13) in root of taspl14 mutant is significantly higher than wild type and the number of companion cells (cluster 21) in root of taspl14 mutant is less than wild type (Fig 5e, 5f). But we are unable to distinguish protophloem from other cells. These results have been included in the revised manuscript (line 298–300) by "The roots of the taspl14 knock-out line showed reduced number of companion cells and increased protoxylem pores in the root transection compared to wild type (Fig. 5e and 5f)". This result is consistent with low expression of BAM 1* *in taspl14 mutant (Fig. 5g). BAM 1 is required for root xylem patterning. It is the downstream target gene of SPL9 (ortholog of TaSPL14) and can repress the protophloem differentiation [**4**,* *5**]. Moreover, we investigated the expression of marker genes of protoxylem (cluster 13), protophloem (cluster 16) and companion cells (cluster 21) in both wild type and the taspl14 mutant. Four out of eight showed significant change of gene expression (Fig. S13). This result has been added into the revised manuscript (line 311–320) as "Moreover, we also investigated the expression of marker genes, including homologs of DA1-RELATED PROTEIN2 (DAR2), LSD ONE LIKE 3 (LOL3), ANNEXIN 5 (ANN5), ALTERED PHLOEM DEVELOPMENT (APL), VND-INTERACTING 2 (VNI2), TARGET OF MONOPTEROS 5 (TMO5), XCP1, and VASCULAR RELATED NAC-DOMAIN PROTEIN 1 (VND1), most of which has been demonstrated to play important roles in protoxylem (cluster 13), protophloem (cluster 16) and companion cells (cluster 21) [**89–94**]. Four out of these eight genes showed statistically significant change in taspl14 knock-out line, including the homologs of LSD ONE LIKE 3 (LOL3) in companion cells, the homologs of ALTERED PHLOEM DEVELOPMENT (APL) [**89**] in protophloem, the homologs of XCP1 [**91**] and VASCULAR RELATED NAC-DOMAIN PROTEIN 1 (VND1) [**90**] in protoxylem (Fig. S13)".*

Fig. 5 (e) Root transections of *taspl14* knock-out line and wild type. The protoxylem pores and companion cells were marked with yellow arrow and pink arrow head, respectively. Bar is 100 μm. (f) *taspl14* knock-out line showed reduced companion cells and increased protoxylem. (g) *BAM1* and *LOB* were down regulated in root of *taspl14* knock-out lines.

Fig. S13 Relative expression of marker genes of companion cells, protophloem and protoxylem in *taspl14* line 5. (* *p*-value < 0.05, ** *p*-value < 0.01, *** *p*-value < 0.001, ns indicate no significant difference).

Minor

- Line 114, "which was comparable with published single cell datasets from Arabidopsis". Which datasets? I could not references.

*Response: Thank you for pointing this out. We are sorry that we put the reference in the Table S1 in the previous version of the manuscript. Now, we have cited the reference in the modified manuscript. Please see line 117 in the revised manuscript.*

- Line 392, what is the D and DW buffer?

*Response: Thank you for the question and sorry for the missed details. We have added the detailed information of D and DW buffer in the modified manuscript as "AK58 in 0.4 M D buffer (0.4 M D-sorbitol, 20 mM 2-Morpholinoethanesulphonic acid, 20 mM KCl, 10 mM MgCl_2_, 0.2% Triton X100, 3 mM Dithiothreitol and 1% Bovine albumin) with RNase inhibitor in it on pre-chilled plates. Resuspend the homogenates with 400 μl 0.4 M DW buffer (0.4 M D-sorbitol, 20 mM 2-Morpholinoethanesulphonic acid, 20 mM KCl, 10 mM MgCl2, 3 mM Dithiothreitol and 1% Bovine albumin)." in line 419–423.*

- Fig. 1c, please use the same code as (b) in the panel.

*Response: Thank you for the suggestion. We have now improved the Fig. 1c.*

Reviewer #2:

This is an interesting study that carries out single cell sequencing for wheat root using both snRNA-seq and snATAC-seq. Especially, the author investigated the asymmetrical transcription for wheat root development. The project provides import key regulators in root development of polyploid wheat.

*Response: Thank you very much for your interests on our work. Wheat has a complex genome that makes functional study in wheat challenging. We wish that the regulatory factors we identified and the cell identities we uncovered would facilitate the root related studies in wheat. Thank you for the comments, questions and suggestions listed below, which inspired us to perform more analysis and the work benefits a lot from the new analysis.*

Bread wheat included the AA, BB and DD genome. Asymmetric expression of homoeologs is important part for this study. However, the short reads were used for snRNA-seq and snATAC-seq in this study. Thus, there are many reads which could be mapped to both AA, BB and DD genome. How did the author calculate the expression of homoeologs? The author should provide detail description or custom pipeline in method section for phasing of reads for snRNA-seq and snATAC-seq to AA, BB and DD genome, respectively.

*Response: Thank you for the comments and suggestions. Indeed, multiple alignment is one of the major challenges for data analysis in wheat when short reads are used to investigate asymmetric expression of homoeologs. To solve the problem, we used poly dT to capture the RNAs in each nucleus and we took the advantage of our nucleus based method to include introns and 3’ UTR, 5’ UTR to count the abundance of RNAs. This greatly reduced the ratio of multiple alignment to homoeologous genes. Our results showed that 73.71% of the total sequenced reads were unique mapped reads (MAPQ = 255, generated by STAR) and 19.94% could be mapped to more than five different genomic regions (MAPQ = 0), which might be due to low quality of the reads or multiple alignment (but not to homoeologs). Only 6.35% of the reads were found to be mapped to 2–4 positions (0 < MAPQ < 255), which could be caused by alignment to homoeologs (usually with 1:1:1 ratio) (Figure R3a). Taken all the above factors into consideration, we set the threshold of MAPQ value to 255 to obtain high quality unique mapped reads. This largely helps to solve the multiple alignment issue. For example, the three homoeologs of TraesCS1A02G023300 / TraesCS1B02G029000 / TraesCS1D02G022800 （D. dominantly expressed in cluster 15） share quite similar exon sequence (Figure R3b) but the majority of the reads are uniquely mapped to each of the members (Figure R3c). And the abundance of TraesCS1D02G022800 is much higher than its homoeologs in AA and BB genomes (Figure R3c and d). For ATAC-seq data analysis, the bwa was used to preform alignment, which would randomly select a mapping position when two or more best alignments occurred. We only reserved the high quality unique mapped reads with MAPQ value higher than 30. We have added the detail parameters for reads alignment in the revised manuscript (Line 455–458) with the sentence of "We used poly dT to capture the RNAs in each nucleus and we took the advantage of our nucleus based* *method to include introns and 3’ UTR, 5’ UTR to count the abundance of RNAs. Only unique mapped reads with MAPQ value of 255 was used to generate a UMI count matrix where each row contains a single gene and each column represents a cell".*

Figure R3 The mapping of snRNA-seq reads on homoeologous genes. (a) The statistics of reads mapped with differnt MAPQ values; (b) The sequence similarity of the three homoeologous triad of *TraesCS1A02G023300*, *TraesCS1B02G029000* and *TraesCS1D02G022800*, the homoeologous triad is D.dominant expressed in cluster 15; (c) The distribution of MAPQ values of reads that mapped to the homoeologous triad; (d) IGV shows the mapped reads of the homoeologous triad.

snRNA-seq can generate higher expression of long noncoding RNAs in snRNA-seq libraries than that of scRNA-seq. The author should provide the expression of long noncoding RNAs in 22 clusters since it is one of the advantage of snRNA-seq method. It will be interesting to find some cluster-specific marker long noncoding RNAs.

*Response: Thank you very much for the suggestion. Following the suggestion, we detected the expression of long noncoding RNAs in the 22 clusters. In general, there are 50–125 long noncoding RNAs identified in each single cluster. Cluster-specific marker long noncoding RNAs were found in cluster 15, 17, 18, 20 and 21. We have included this information in the Fig. S4, Table S3 and line 142–145 as "We then investigated the expression of long noncoding RNAs in the 22 clusters. 50–125 long noncoding RNAs were identified in the particular cluster. Interestingly, we found cluster-specific marker long noncoding RNAs for cluster 15, 17, 18, 20 and 21 (Fig. S4 and Table S3)." and we have revised the method part accordingly in line 489–497.*

Fig. S4 Expression of long noncoding RNAs in each cluster. (a) Number of expressed long noncoding RNAs in each cluster. (b) Representative long noncoding RNAs of each cluster. (c) Heatmap showing the cluster enriched long noncoding RNAs in each cell clusters.

Line 135 The author can compare the 22 cluster-enriched genes from snRNA-seq in this study with that from published scRNA-seq/snRNA-seq root data in other species to present the percentage of overlapped orthologous genes for each cluster.

*Response: Thank you for the insightful suggestion. We performed pairwise comparisons of root cell clusters in Arabidopsis, rice and wheat [**1**,* *3**].(In the maize root single cell paper, the author said there are 21 cell clusters but the data showed that there are 22 clusters. So, we skipped maize in this analysis) [**2**]. The clusters of meristem, root hair, phloem, xylem and root cap are quite similar between Arabidopsis and wheat* *(Fig. 2i). Rice and wheat share similar marker gene in the clusters of endodermis, epidermis, meristem, phloem (phloem, companion cell and sieve element) and xylem (Fig. 2j). Higher percentage of overlapped orthologous genes were found in meristem, phloem and xylem between species (Fig. 2k). These results may indicate a relative conserved regulation for meristem, phloem and xylem development among species. We have included this information in the revised manuscript. Please see Fig. 2i, 2j, 2k and line 175–181. "We further performed pairwise comparisons of root cell clusters identified by the scRNA-seq/snRNA-seq data in Arabidopsis, rice and wheat (Fig. 2i-2k) [**21**,* *22**]. The marker genes for clusters of endodermis, epidermis (root hair and no root hair), meristem, phloem (phloem, companion cell and sieve element) and xylem showed a relatively high similarity in rice and wheat (Fig. 2j). The Sankey diagram showed significantly higher percentage of overlapped orthologous genes for meristem, phloem and xylem between species (Fig. 2k). These results may suggest a conserved regulation network for meristem, phloem and xylem development in plant." and we have revised the method part accordingly in line 498–504.*

Fig. 2 (i) Pairwise correlations of *Arabidopsis* (left) and wheat (top) root cell clusters. Dots indicate statistically significant correlations. (j) Pairwise correlations of rice (top) and wheat (left) root cell clusters. Dots indicate statistically significant correlations. (k) Sankey diagram showed that wheat shares a high degree of similarities in epidermal cell (root hair and no root hair), meristem, phloem (phloem, companion cell and sieve element) and xylem with rice and *Arabidopsis*. The number of overlapped orthologous genes for each cell type is given on the right of each cluster.

Line 136-138 The authors should provide expression information (heatmap) of more top enriched genes rather than only several representative cluster-specific marker genes (Fig. 1d) of 22 clusters.

*Response: Thank you for the suggestion. We have now presented the top10 enriched genes in the 22 clusters and each cell cluster is clearly distinguished from other cell clusters. Please see Fig. 1d and line 141–142. The sentence in line 141–142 is changed into "The top 10 cluster-specific marker genes with specific expression pattern were shown in Fig. 1d."*

Fig. 1 (d) The expression patterns of representative top 10 cluster-specific marker genes in 22 clusters.

Line 252 It's great interesting to see that snATAC-seq correlated with snRNA-seq. However, the result about snATAC-seq part is weak. The authors only provided general profile information for the association. Did the important cluster-specific marker genes present the association? Thus, more case study information should be provided for each cluster-specific marker genes to deeply mine snATAC-seq information.

*Response: Thank you for the positive comments and the nice suggestion. Following the suggestion, we analyzed the cluster-specific marker genes identified by snATAC-seq. The common cluster specific marker genes between snATAC-seq and snRNA-seq were shown in the revised manuscript. Please see Fig. S9. We* *also scanned for the representative cis-motifs in each cluster. Please see Fig. S10 and line 266–270 as "Cluster specific marker genes presented in snRNA-seq UMAP plots also showed cluster specific pattern in snATAC-seq UMAP plots, which indicated high relevance between the snRNA-seq and snATAC-seq dataset (Fig. S9). The cluster representative cis-motifs were also identified by using snATAC-seq data (Fig. S10)".*

*As a case study, we found that the distal region and transcription start site (TSS) of BAM1 gene were highly accessible in protoxylem, metaxylem and meristem but not in other cell clusters. BAM1 has been identified as the target gene of TaSPL14 to regulate the protoxylem, companion cell and protophloem and we found the binding motif of SPL14 in the ATAC-seq peak. Please see Fig. 5h and line 305–309 as "The chromatin located at upstream of BAM1 homologs (chr4D_part1:397107039–397107426 and TraesCS4D02G235800) was only specifically accessible in protoxylem, metaxylem and meristem and the binding motifs of TaSPL14 were enriched in this region. Interestingly, we also detected the binding of TaSPL14 to this region by analyzing a published DAP-seq data [**88**] (Fig. 5h)".*

Fig. S9 UMAP plots showing cluster specificity of common marker genes between the corresponding clusters of snRNA-seq and snATAC-seq.

Fig. S10 Representative motifs for each cluster of snATAC-seq.

Fig. 5(h) The accessible chromatin regions (ACRs) of *BAM1* homologs (*TraesCS4D02G235800*).

The author should provide an online web server to facilitate the usage of the snRNA-seq and snATAC-seq datasets generated in this manuscript.

*Response: Thank you for the suggestion and it is indeed a great idea and. We have now provided the online web server for the users to visualize the expression level and accessibility of each gene in each cell. The web server consistent of four parts, namely introduction, snRNA, snATAC and snATAC mapping to snRNA. The user only needs to submit a gene ID to the snRNA webpage and then the information of the expression levels in each cell visualized by UMAP, t-SNE, violinplot and dotplot could be easily obtained. By submitting a gene ID to the snATAC webpage, the gene score, chromatin accessibility, co-accessible peaks and peak to gene links could be visualized by UMAP or browser track. In addition, all the marker genes, gene average expression and peaks to gene links could be download from the snATAC mapping to snRNA part. The link of the online web server is* *"http://crispr.hzau.edu.cn/wheataltas/html/destinations.html".* *Please see line 96–98 as "An online web server (http://crispr.hzau.edu.cn/wheataltas/html/destinations.html) was also provided for user to easily use and visualize the snRNA-seq and snATAC-seq datasets generated in this study".*

*Reference*

*1.	Zhang TQ, Xu ZG, Shang GD, Wang JW. A single-cell RNA sequencing profiles the developmental landscape of Arabidopsis root. Mol Plant. 2019;12:648–660.*

*2.	Ortiz-Ramirez C, Guillotin B, Xu X, Rahni R, Zhang S, Yan Z, et al: Ground tissue circuitry regulates organ complexity in maize and Setaria. Science. 2021;374:1247–1252.*

*3.	Zhang TQ, Chen Y, Liu Y, Lin WH, Wang JW. Single-cell transcriptome atlas and chromatin accessibility landscape reveal differentiation trajectories in the rice root. Nat Commun. 2021;12:2053.*

*4.	Hu C, Zhu Y, Cui Y, Zeng L, Li S, Meng F, et al. A CLE-BAM-CIK signalling module controls root protophloem differentiation in Arabidopsis. New Phytol. 2022;233:282–296.*

*5.	Fan P, Aguilar E, Bradai M, Xue H, Wang H, Rosas-Diaz T, et al. The receptor-like kinases BAM1 and BAM2 are required for root xylem patterning. Proc Natl Acad Sci U S A. 2021; 118(12):e2022547118.*

*6.	Pei H, Teng W, Gao L, Gao H, Ren X, Liu Y, et al. Low-affinity SPL binding sites contribute to subgenome expression divergence in allohexaploid wheat. Sci China Life Sci. 2022. https://doi.org/10.1007/s11427-022-2202-3.*

**Second round of review**

**Reviewer 1**

Authors have addressed my concerns and I am happy to recommend it for publication in Genome Biol.

**Reviewer 2**

Thanks the authors for their efforts to improve the manuscript. The authors have addressed all my comments in the revised manuscript. The manuscript has been significantly improved.
